# Comprehensive discovery and functional characterization of the noncanonical proteome

**DOI:** 10.1038/s41422-024-01059-3

**Published:** 2025-01-10

**Authors:** Chengyu Shi, Fangzhou Liu, Xinwan Su, Zuozhen Yang, Ying Wang, Shanshan Xie, Shaofang Xie, Qiang Sun, Yu Chen, Lingjie Sang, Manman Tan, Linyu Zhu, Kai Lei, Junhong Li, Jiecheng Yang, Zerui Gao, Meng Yu, Xinyi Wang, Junfeng Wang, Jing Chen, Wei Zhuo, Zhaoyuan Fang, Jian Liu, Qingfeng Yan, Dante Neculai, Qiming Sun, Jianzhong Shao, Weiqiang Lin, Wei Liu, Jian Chen, Liangjing Wang, Yang Liu, Xu Li, Tianhua Zhou, Aifu Lin

**Affiliations:** 1 The Center for RNA Medicine, International Institutes of Medicine, International School of Medicine, The 4th Affiliated Hospital of Zhejiang University School of Medicine, Yiwu, Zhejiang China; 2 MOE Laboratory of Biosystem Homeostasis and Protection, College of Life Sciences, Zhejiang University, Hangzhou, Zhejiang China; 3 Cancer Center, Zhejiang University, Hangzhou, Zhejiang China; 4 Key Laboratory of Cancer Prevention and Intervention, China National Ministry of Education, Hangzhou, Zhejiang China; 5 Department of Cell Biology and Program in Molecular Cell Biology, Zhejiang University School of Medicine, Hangzhou, Zhejiang China; 6 Department of Gastroenterology, the Second Affiliated Hospital, School of Medicine and Institute of Gastroenterology, Zhejiang University, Hangzhou, Zhejiang China; 7 Key Laboratory of Structural Biology of Zhejiang Province, Westlake Laboratory of Life Sciences and Biomedicine, Westlake University, Hangzhou, Zhejiang China; 8 Department of Gastrointestinal Surgery, The Second Affiliated Hospital, Zhejiang University School of Medicine, Hangzhou, Zhejiang China; 9 Zhejiang University-University of Edinburgh Institute, Zhejiang University School of Medicine, Haining, Zhejiang China; 10 The Second Affiliated Hospital, Zhejiang University School of Medicine, Hangzhou, Zhejiang China; 11 Hangzhou Cancer Hospital, Hangzhou, Zhejiang China; 12 Department of Nephrology, Center for Regeneration and Aging Medicine, The Fourth Affiliated Hospital of School of Medicine and International School of Medicine, International Institutes of Medicine, Zhejiang University, Yiwu, Zhejiang China; 13 Institute of Immunology, Zhejiang University School of Medicine, Hangzhou, Zhejiang China; 14 Department of Molecular Genetics, University of Toronto, Toronto, ON Canada; 15 Future Health Laboratory, Innovation Center of Yangtze River Delta, Zhejiang University, Jiashan, Zhejiang China; 16 Key Laboratory for Cell and Gene Engineering of Zhejiang Province, Hangzhou, Zhejiang China

**Keywords:** Molecular biology, Cell biology, Cancer

## Abstract

The systematic identification and functional characterization of noncanonical translation products, such as novel peptides, will facilitate the understanding of the human genome and provide new insights into cell biology. Here, we constructed a high-coverage peptide sequencing reference library with 11,668,944 open reading frames and employed an ultrafiltration tandem mass spectrometry assay to identify novel peptides. Through these methods, we discovered 8945 previously unannotated peptides from normal gastric tissues, gastric cancer tissues and cell lines, nearly half of which were derived from noncoding RNAs. Moreover, our CRISPR screening revealed that 1161 peptides are involved in tumor cell proliferation. The presence and physiological function of a subset of these peptides, selected based on screening scores, amino acid length, and various indicators, were verified through Flag-knockin and multiple other methods. To further characterize the potential regulatory mechanisms involved, we constructed a framework based on artificial intelligence structure prediction and peptide‒protein interaction network analysis for the top 100 candidates and revealed that these cancer-related peptides have diverse subcellular locations and participate in organelle-specific processes. Further investigation verified the interacting partners of pep1-nc-OLMALINC, pep5-nc-TRHDE-AS1, pep-nc-ZNF436-AS1 and pep2-nc-AC027045.3, and the functions of these peptides in mitochondrial complex assembly, energy metabolism, and cholesterol metabolism, respectively. We showed that pep5-nc-TRHDE-AS1 and pep2-nc-AC027045.3 had substantial impacts on tumor growth in xenograft models. Furthermore, the dysregulation of these four peptides is closely correlated with clinical prognosis. Taken together, our study provides a comprehensive characterization of the noncanonical proteome, and highlights critical roles of these previously unannotated peptides in cancer biology.

## Introduction

The sequencing of the human genome by the Human Genome Project has greatly contributed to a genome-wide understanding of complex biological processes.^1^ The annotation of protein-coding regions in the genome is an essential component of functional genomics and contributes to a deeper understanding of proteins.^2^ However, only approximately 1% of the genome is composed of protein-coding regions. The majority of the remaining genome consists of noncoding regions that produce abundant noncoding RNAs (ncRNAs), such as long noncoding RNAs (lncRNAs).^3,4^ Previous studies, including our own, reported that lncRNAs could modulate multiple cellular activities and cancer progression.^5–11^ In addition, lncRNA transcripts share polyadenylation and capping characteristics with mRNA transcripts.^12^ In recent years, a series of studies have shown that lncRNAs have the potential to encode novel peptides. For example, Anderson et al. reported that myoregulin (MLN), a peptide encoded by the lncRNA *LINC00948*, plays a key role in skeletal muscle physiological function,^13^ drawing attention to the identification and function investigation of the products encoded by individual short open reading frames (sORFs). There is mounting evidence that these individual sORF products play important roles in multiple cellular processes, including signaling regulation,^14–16^ metabolic homeostasis,^17–21^ RNA splicing and modification recognition,^22,23^ transcription and translation regulation,^24,25^ and the immune response.^26,27^ Their precise and limited expression is vital for normal physiological function, and deviations in their expression levels can lead to various issues, including impaired muscle function,^13,14^ obesity,^17,18,28^ inflammatory diseases,^26,27^ and cancer.^15,16,20,22–25^ Therefore, it is important to systematically identify and functionally characterize sORFs and novel peptides in the genome to gain new insights into the genome, cells, and specific diseases.

The process of identifying novel peptides and sORFs relies on technological advancements. Bioinformatics formed the foundation of the identification in the preliminary stages to predict novel peptides and sORFs. Cabili et al. discovered that highly conserved long intergenic noncoding RNA transcripts may be potential sources of sORFs by analyzing RNA-seq data.^29^ Various computational tools, such as coding-potential assessment tool (CPAT),^30^ have been developed, and specific peptides can be identified by computational prediction,^31–33^ providing a preliminary reference for spectrum matching in mass spectrometry (MS). Banfai et al. and Slavoff et al. successfully identified nearly 100 novel sORFs in human cell lines through the integration of RNA-seq and MS data^34^ or peptidomic strategies.^35^ This approach has evolved into a primary strategy for identifying novel peptides by MS, fortified by advancements such as non-ATG start codon identification^36^ and specific amino acid enrichment strategies.^37^ Additionally, novel peptides have been unintentionally discovered during conventional proteomics studies using database such as ProteomicsDB.^2^ Nevertheless, challenges associated with novel peptides, such as short sequences, low abundance, and the absence of comprehensive reference databases and targeting strategies, have constrained the identification of novel peptides; the number of identified peptides typically ranges from tens to hundreds, with few studies exceeding 1000 in large sample sizes.^2^

In light of the constrained precision of MS for the identification of novel peptides, the application of new technologies has emerged as a pivotal breakthrough. Ribo-seq, which employs PCR amplification and RNA-seq, has proven to be suitable for the identification of sORFs with low translational intensity, providing a vital technical support for systematical identification of sORFs.^38,39^ Zhang et al. completed the pioneering work of applying Ribo-seq to identify lncRNAs with translation potential on human chromosome 1,^40^ and Bazzni et al. further extended its utility for novel peptide discovery in zebrafish during embryogenesis.^41^ Modifications by Ingolia et al. have improved the precision of recognizing ribosome footprints outside the coding region,^42^ facilitating the determination of translation of uORFs (upstream ORFs), dORFs (downstream ORFs) and lncRNAs in human cell lines. A series of studies applied Ribo-Seq to a wide range of cell lines and the translation maps of them were systematically constructed.^43–49^ The large-scale identification of sORFs at the tissue level has also subsequently been achieved. Van Heesch et al. successfully mapped the translational landscape of the human heart by Ribo-seq and revealed a substantial number of lncRNAs with translational potential.^47^ Subsequent endeavors have concentrated on the efficient identification of sORFs while achieving high-throughput targeted functional characterization. Chen et al. successfully applied CRISPR technology to screen for functional novel peptides at the cell line level.^48^ Martinez et al. further carried out CRISPR screening in animal models and identified novel peptides with metabolic regulatory functions.^28^ The analysis of the properties of these peptides has been substantially advanced by the development of bioinformatics frameworks and molecular biology tools targeting novel peptides. Noteworthy examples include the evolutionary analysis framework for sORFs constructed by Sandmann et al.,^50,51^ the bioinformatics pipeline for sORF homolog detection by Patraquim et al.,^52^ and the MicroID tool for subcellular localization screening of novel peptides by Na et al.^53^

Owing to these technological strides, the field of novel peptidomics has experienced substantial advancements. Nevertheless, challenges persist. Ribo-seq relies on RNA-seq, and primarily detects RNA fragments during translation. While informative at the RNA level, it falls short in providing direct evidence of novel peptides at the proteomic level, which remains the gold standard for the identification of novel proteins. Although some studies have used MS in combination with other strategies, the number of identified peptides remains limited, and only a few hundred peptides can be identified in most cases.^47,48,54^ Besides, progress in the development of current MS strategies has been sluggish. While immunopeptidomics, a subtechnique of MS, has revealed a greater abundance of novel peptides with immunogenicity, their functions remain to be fully characterized,^55–57^ casting doubt on the authenticity of these peptides as functional peptides. The frontier lies in integrating identification, biological functional characterization, and physiological phenotyping. However, studies that successfully integrate these elements are still limited.

Gastric cancer, ranked as the fifth most prevalent cancer globally, is distinguished by its high heterogeneity and the absence of early diagnostic markers and targeted therapies.^58,59^ Recent genomic, transcriptomic, and proteomic studies have exposed the multi-omic characteristics of gastric cancer and identified new diagnostic and therapeutic targets.^60–63^ Despite these advancements, systematic studies of novel peptidomics in gastric cancer remain unexplored. Previous research has hinted at the potential applications of peptides, such as ProGRP, in managing gastric cancer.^64^ Systematic investigations into novel peptidomics could facilitate our understanding of gastric cancer, potentially offering fresh perspectives for diagnosis and treatment by exploring peptides with key regulatory functions.

In our study, we addressed the aforementioned challenges through several key advancements. First, we reassembled and mined the latest human transcriptome. This effort resulted in the creation of a reference database covering 11,668,944 potential sORFs. To address issues in identifying these peptides with their low abundance and short sequences, we employed an ultrafiltration tandem MS assay. By integrating these two techniques, we successfully identified 8945 previously unannotated novel peptides in human gastric cancer samples and cell lines, marking the largest number of novel peptides ever identified at the proteomic level. We further pinpointed 1161 candidate peptides that were closely associated with cell proliferation by CRISPR screening in AGS cells. Moreover, we used molecular biology techniques such as Flag-knockin to confirm the authenticity and biological importance of these functional peptides. Building upon this foundation, we established a peptide function prediction framework based on AlphaFold2 structure prediction and peptide‒protein interaction network construction. This framework revealed the diverse subcellular localizations of these peptides and their functional mechanisms, particularly those related to complex assembly and energy metabolism. Subsequent validation in xenograft tumor models and patient clinical samples demonstrated that the tested peptides regulated tumor growth in vivo and were strongly correlated with patient clinical prognosis. Our work represents a notable attempt at high-throughput peptidomics research, encompassing technological improvements, large-scale tissue-level identification, functional screening, phenotypic characterization, and clinical relevance studies. This collective effort highlights the importance of exploring and characterizing the novel peptidome as a promising approach for advancing tumor diagnostic and treatment strategies.

## Results

### Identification of novel peptides using bioinformatics analysis and ultrafiltration tandem MS

A key obstacle in the proteomic identification of novel peptides is the absence of a comprehensive, high-precision peptide sequencing reference library. To overcome this, we utilized human transcript data from Ensembl to reassemble the reference transcriptome and extract all potential ORFs that contain start/stop codons using Ribotricer.^65^ Since novel peptides are often short and derived from RNAs with noncanonical start codons,^66,67^ we filtered the extracted ORFs whose lengths < 250 amino acids and allowed any of ATG/CTG/GTG/TTG as start codons. This yielded 11,668,944 ORFs, which we designated as the reference library of novel peptide ORFs (RLNPORF) for the human genome. Compared to other peptide ORF reference library, RLNPORF includes a larger number of theoretical ORFs and more peptides can be detected using RLNPORF under the same search conditions^68–70^ (Supplementary information, Fig. S1a, b).

**Fig. 1:**
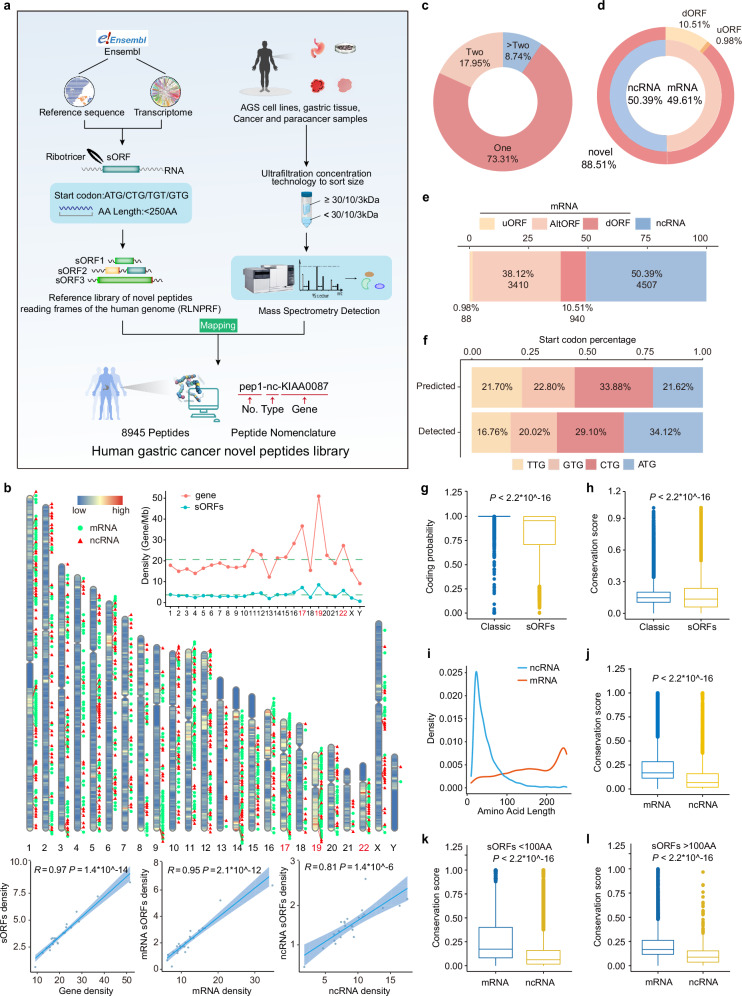
The identification of the novel peptidomes. **a** Schematic overview of the peptide identification process. **b** Distribution of sORFs and genes on chromosomes (upper). The relative quantity relationships between sORF and gene (lower left), mRNA-derived sORF and mRNA gene (lower median), ncRNA-derived sORF and ncRNA gene (lower right) on each chromosome were shown. Each dot represents a chromosome. The correlations were characterized by the spearsman correlation coefficient. **c** Sunburst map of the genes grouped by the number of peptides they encode. **d** Sunburst map of sORFs grouped by the host transcript type of them. uORF: upstream ORFs; dORF: downstream ORFs. **e** Stacked bar plot for number and percentage of detected sORFs. **f** Start codon (ATG/CTG/GTG/TTG) usage of peptides in the RLNPORF vs those actually identified by MS. **g** Boxplot for coding potential score comparison of ORFs encoding classic proteins (included in Uniprot) and sORFs analyzed by CPAT. Data are presented as individual value and statistic analysis was performed using two-sided unpaired two-samples non-parametric Wilcoxon test, *P* < 2.2E−16. **h** Boxplot for conservation score comparison of ORFs encoding classic proteins (included in Uniprot) and sORFs analyzed by Phastcons. Data are presented as individual value and statistic analysis was performed using two-sided unpaired two-samples non-parametric Wilcoxon test, *P* < 2.2E−16. **i** Density map for amino acid length of sORF derived from ncRNA or mRNA. **j** Boxplot for conservation score comparison of sORFs derived from mRNA and ncRNA analyzed by Phastcons. Data are presented as individual value and statistic analysis was performed using two-sided unpaired two-samples non-parametric Wilcoxon test, *P* < 2.2E−16. **k** Boxplot for conservation score of sORFs < 100 aa derived from mRNA and ncRNA analyzed by Phastcons comparison. Data are presented as individual value and statistic analysis was performed using two-sided unpaired two-samples non-parametric Wilcoxon test, *P* < 2.2E−16. **l** Boxplot for conservation score comparison of sORFs > 100 aa and < 250 aa derived from mRNA and ncRNA analyzed by Phastcons. Data are presented as individual value and statistic analysis was performed using two-sided unpaired two-samples non-parametric Wilcoxon test, *P* < 2.2E−16.

We employed liquid chromatography-tandem mass spectrometry (LC-MS/MS), the gold standard for novel peptide/protein identification, to experimentally validate novel peptide candidates from RLNPORF. Sample inputs included 6 pairs of cancer/paraneoplastic tissues, 5 normal gastric tissues, and AGS gastric cancer cell lines. Previous research has suggested that ultrafiltration may be more effective in enriching novel peptide signals while minimizing interference from high molecular weight conventional proteins.^71–73^ Following experimental validation, we identified the optimal treatment strategy: milled and lysed samples were sequentially processed through 30/10/3 kDa ultrafiltration tubes for 20 min (Supplementary information, Fig. S1c, d). The results were consistent across replicates, demonstrating that the strategy can be reliably employed for sample processing. MS spectra were mapped to RLNPORF peptide sequences, and any spectra matching multiple distinct peptides were discarded to ensure confident assignments. We identified 8945 peptides that were not yet included in UniProtKB_Reviewed database (Fig. 1a; Supplementary information, Fig. S1e and Tables S1, S2).

Given the substantial number of peptides identified and their diverse originating gene types, we devised a standardized nomenclature system to effectively distinguish these peptides and access their genomic information. Each peptide is named using the format “pepNo.-Type-Gene”, where “No.” is a numbering order based on the genomic coordinates of the parent ORF, “Type” indicates the class of ORF it derives from (u: upstream ORF (uORF); alt: alternative ORF; d: downstream ORF (dORF); nc: ncRNA), and “Gene” is the gene/locus from which the peptide originates. If a gene encodes only one identified peptide, it is simply labeled as “pep” without numbering. In addition, each peptide receives a unique “ORF ID” that precisely specifies the genomic coordinates of its ORF on the parent transcript, including the transcript ID, ORF start coordinate, ORF stop coordinate, and ORF length in nucleotides. For example, “pep1-nc-KIAA0087” denotes the first peptide identified from the ncRNA KIAA0087 locus, with the ORF ID “ENST00000242109_26533546_26533680_135” indicating that it derives from the transcript ENST00000242109 with an ORF spanning genomic coordinates 26533546-26533680 and a length of 135 nucleotides.

In our peptide identification, 4097 (45.6%) peptides were supported by single peptide-spectrum match (PSM) and 4866 (54.4%) peptides were supported by at least two different PSMs (Supplementary information, Figs. S1f) and 2290 (25.6%) peptides were detected across different tissue/cell line samples (Supplementary information, Fig. S1g). Furthermore, analysis against an alternative human genome reference (CN1)^74^ matched 8823 of the identified sORFs (98.63% coverage), confirming the authenticity of these peptides at the transcriptome level (Supplementary information, Fig. S1h).

In summary, we have successfully implemented a proteomics strategy tailored for identifying peptides by leveraging an approximately  12 million entry reference library of potential ORFs combined with an ultrafiltration-based MS enrichment approach. This integration enabled the identification of nearly 9000 peptides across real-world sample types, representing a valuable resource for furthering our understanding of noncanonical translation products.

### Bioinformatic characterization of the novel peptidome

To gain insights into the properties of these newly identified peptides, we performed bioinformatic analyses comparing them to known proteins from the conventional proteome.

We first examined the chromosomal distribution of genes encoding these peptides. Similar to “regular” genes, the novel peptide genes were most densely localized to chromosomes 17, 19, and 22 (Fig. 1b). The majority of genes produced only a single detected novel peptide, while a small subset generated multiple novel peptide products (Fig. 1c). Interestingly, of these newly identified peptides, 88.51% were translated from novel ORFs and 50.39% were derived from ncRNAs (Fig. 1d, e), suggesting a vastly underappreciated coding potential in the human genome.

Although there was no substantial preference for the four start codons (ATG/CTG/TTG/GTG) by peptides in the reference library, sequence analysis revealed that the identified peptides still showed a preference for the canonical ATG (34.12%) start codon (Fig. 1f). And the peptides we identified generally had lower CPAT^75^ scores and vertebrate conservation compared to known proteins included in Uniprot (Fig. 1g, h), which may explain why they evaded previous annotation efforts.

We further characterized the subset of peptides originating from ncRNAs. The peptides derived from ncRNAs were shorter than those from mRNAs, with most being less than 100 aa (Fig. 1i). The peptides derived from ncRNAs were comparable in length to uORF peptides (Supplementary information, Fig. S1i) but exhibited lower sequence conservation (Fig. 1j; Supplementary information, Fig. S1j). They exhibited lower conservation scores and coding probability than those derived from mRNAs, regardless of length. The difference in coding probability between these two groups is more pronounced for peptides < 100 aa (Fig. 1k, l; Supplementary information, Fig. S1k‒m). Importantly, a significant proportion of the novel peptide sequences matched known protein domains^76^ (Supplementary information, Fig. S1n), implying functional potential. Collectively, these findings reveal that ncRNAs can produce functional peptides that differ from conventional proteins in their sequence characteristics.

Finally, as these peptides were identified from gastric cancer-related tissues and cells, we assessed potential links between these peptides and gastric cancer pathogenesis by analyzing their host gene (6216) expression patterns. Strikingly, the expression of 994 (16.0%) novel peptide genes showed associations with histopathological features of gastric cancer: 795 (12.8%) with pathological stage, 797 (12.8%) with pathological subtype specificity, and 329 (5.3%) with chemotherapy resistance (Supplementary information, Fig. S2a‒d and Tables S3‒S6). These correlations suggest prominent roles for the newly identified peptides in regulating gastric cancer progression and disease states.

In summary, this integrated bioinformatic characterization highlights the unique properties of the newly uncovered peptidome, including peptide genomic origins, sequence traits, and potential functional impacts, particularly in gastric cancer pathogenesis. These findings set the stage for deeper mechanistic investigations into novel peptide biology and its therapeutic applications.

### Evaluation of the roles of peptides in cancer cell proliferation via CRISPR screening

To complement the proteomic evidence, we employed a high-throughput CRISPR screening^77^ approach to directly evaluate the functional impacts of these peptides.

**Fig. 2:**
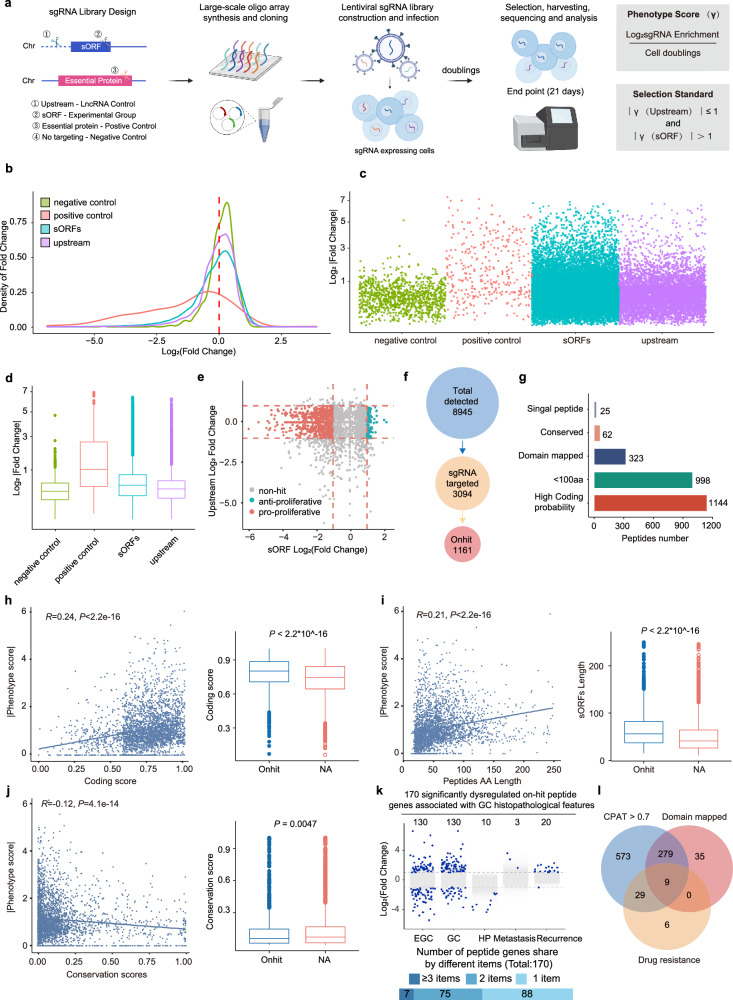
CRISPR screening reveals key role of peptides in the regulation of cell proliferation. **a** Schematic map of CRISPR screening process for peptides. **b** Density map depicting the absolute values of fold changes for sgRNAs targeting essential proteins, scrambled sequences, sORFs, and regions upstream of sORFs.  **c**, **d** Jitter (**c**) and box (**d**) plots depicting the absolute values of fold changes for sgRNAs targeting essential protein, scramble sequence, sORFs and upstream of sORFs. **e** Point plot for the screening results of peptides. Red points indicate pro-proliferative peptides, green points indicated anti-proliferative peptides, and gray points indicated non-hit peptides. The criterion was defined as |log_2_fold change of sORFs body | > 1 and |log_2_fold change of sORFs upstream | < 1. **f** Statistic of overall detected peptides (8945), peptides designed for CRISPR screening (3094) and phenotypic peptides (1161). **g** A tally of on-hit peptides with specific genetic or biochemical properties. **h** Correlation between the absolute values of phenotype score and coding score of peptides analyzed by CPAT. The correlations were characterized by the spearsman correlation coefficient (left). Boxplot for coding potential score comparison of phenotypic and non-phenotypic peptides (right). Wilcoxon test; *P* < 2.2E−16. **i** Correlation between the absolute values of phenotype score and peptide amino acid length. The correlations were characterized by the spearsman correlation coefficient (left). Boxplot for peptide amino acid comparison of phenotypic and non-phenotypic peptides (right). Wilcoxon test; *P* < 2.2E−16. **j** Correlation between the absolute values of phenotype score and conservation score analyzed by Phastcons. The correlations were characterized by the spearsman correlation coefficient (left). Boxplot for conversation score comparison of phenotypic and non-phenotypic peptides (right). Wilcoxon test; *P* = 0.0047 E. **k** Upper: Manhattan map of the peptide genes associated histopathological feature of gastric cancer. Cutoff threshold of significantly changed genes was defined as |log_2_fold change | > 1 and adjusted *P* value < 0.05. Data were presented as individual log_2_fold change value. Lower: The number of peptide genes shared by different items. **l** Venn diagram showing peptides with high coding probability (CPAT > 0.7), domain mapped and association with drug resistance.

To avoid confounding effects of conventional protein isoforms, we focused on the 4507 lncRNA-derived and 88 uORF-derived peptides for a genome-scale CRISPR library design. As the design criteria, each putative peptide ORF should be able to be targeted by at least 4 single guide RNAs (sgRNAs), with an additional ≥ 2 sgRNAs targeting 1000 bp upstream of its ORF as host lncRNA knockout (KO) controls. This yielded a library of 27,113 sgRNAs covering 3094 peptides, along with 1041 non-targeting scramble sgRNAs and 344 sgRNAs against genes essential for cancer cell proliferation as controls. Pooled CRISPR KO screens were performed in the AGS gastric cancer cell line and phenotype score for each peptide was designated as the average log_2_fold change in abundance of sgRNAs targeting that peptide after 10 doublings (Fig. 2a).

The fold change in sgRNA abundance differed significantly between the scramble, essential gene, peptide ORF, and host lncRNA KO groups, validating the feasibility of screening (Fig. 2b). Notably, sgRNAs directly targeting the peptide ORFs exhibited higher overall fold change in abundance and a greater proportion above the significance cutoff (log_2_|fold change | ≥ 1) compared to scramble sgRNA and sgRNA targeting upstream of the ORFs (Fig. 2c, d). This implicates specific regulatory roles for the peptides themselves beyond effects of their host lncRNAs. We also categorized the peptides by their phenotype scores: peptides with scores ≥ 1 were deemed anti-proliferative, while scores ≤ ‒1 indicated pro-proliferative properties. Using these classifications, we identified 1161 peptides with significant impacts on cell proliferation, with nearly 90% demonstrating a pro-proliferative phenotype (Fig. 2e, f; Supplementary information, Table S7). We analyzed the sequences of these peptides, identifying some with signal peptides or functional domains. Additionally, most are predicted to have high coding probability, although only a few exhibit sequence conservation across vertebrates (Fig. 2g).

Through further sequence comparison with RLNPORF, we noticed that there were 1280 predicted ORFs without MS evidence that were targeted by sgRNAs from the lncRNA KO control group (Supplementary information, Fig. S3a). Notably, the fold change in abundance of these sgRNAs was similar to that of scramble sgRNAs (Supplementary information, Fig. S3b, c), indicating that those predicted peptides without MS evidence were unverifiable at the functional proteome level. These findings further underscored the accuracy of our peptide MS identification strategy.

Furthermore, we analyzed the associations between phenotype scores of peptides and their genetic properties. Peptides that regulate tumor growth tend to have higher coding potential scores and be longer than those lacking phenotypes (Fig. 2h, i). Interestingly, peptides that regulated tumor growth exhibited lower sequence conservation, suggesting recent evolutionary origins (Fig. 2j). Additionally, peptides were derived from a total of 1071 genes. Among these, 170 (16.0%) of them were associated with histopathological features, 60 (5.6%) were associated with pathological stage, 65 (6.1%) were associated with subtype specificity, and 35 (3.3%) were associated with chemotherapy resistance in gastric cancer (Fig. 2k, l; Supplementary information, Fig. S3d‒f and Tables S8‒S11), pointing toward their pathogenic potential.

In summary, this unbiased CRISPR screening provided functional evidence for these peptides, uncovering that over 1000 peptides impact cancer cell proliferation. Integrated with the bioinformatic analyses, these results highlight promising candidates for more in-depth mechanistic studies into their biological functions and cancer relevance.

### Verification of functional peptides identified by CRISPR screening

**Fig. 3:**
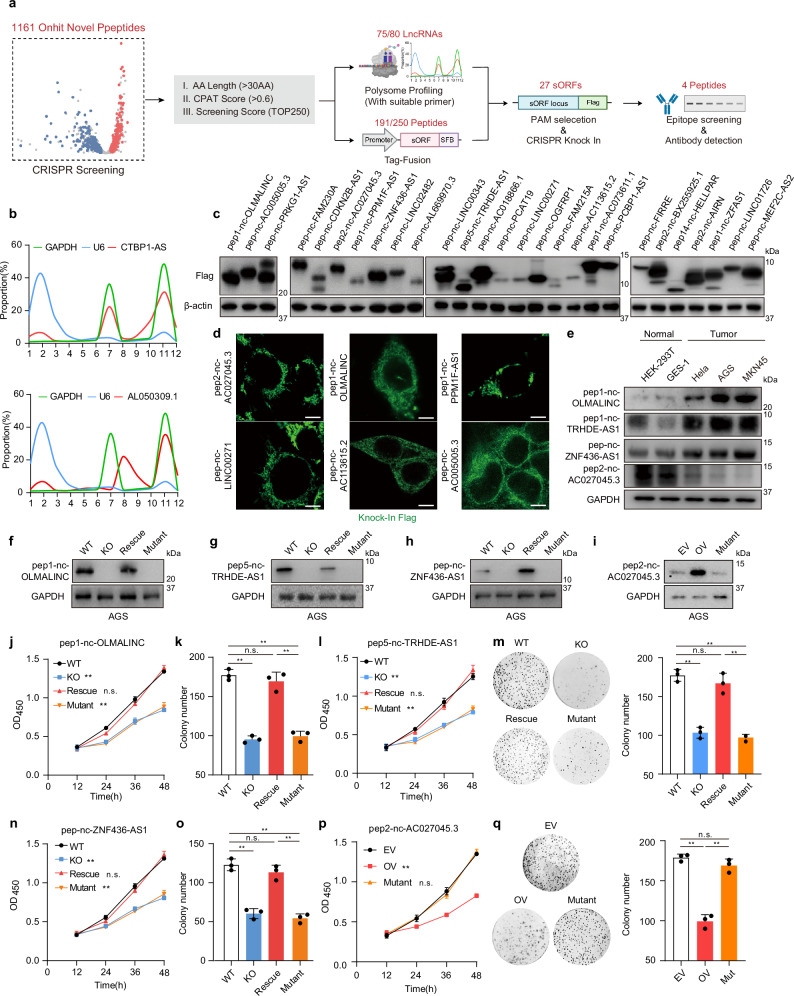
Deep illustration of functional peptides identified by CRISPR screening. **a** Schematic diagram of multiple molecular experimental assays for peptide validation. **b** Representative polysome profiling results of CTBP1-AS, AL050309.1, U6 and GAPDH in AGS cell lines. **c** Immunoblots of peptides (Flag-knockin) in AGS cells. **d** Peptides (Flag-knockin) in AGS cells were detected by immunofluorescence staining. **e** Immunoblots of pep1-nc-OLMALINC, pep5-nc-TRHDE-AS1, pep-nc-ZNF436-AS1, pep2-nc-AC027045.3 and GAPDH in HEK-293T, HeLa, GES-1, MKN45 and AGS cells. **f** Immunoblot analysis in the WT, pep1-nc-OLMALINC KO, pep1-nc-OLMALINC KO-WT ORF back-complemented (Rescue), pep1-nc-OLMALINC KO-start codon mutant ORF back-complemented (Mutant) AGS cells. **g** Immunoblot analysis in the WT, pep5-nc-TRHDE-AS1 KO, pep5-nc-TRHDE-AS1 KO-WT ORF back-complemented (Rescue), pep5-nc-TRHDE-AS1 KO-start codon mutant ORF back-complemented (Mutant) AGS cells. **h** Immunoblot analysis in the WT, pep-nc-ZNF436-AS1 KO, pep-nc-ZNF436-AS1 KO-WT ORF back-complemented (Rescue), pep-nc-ZNF436-AS1 KO-Start codon mutant ORF back-complemented (Mutant) AGS cells. **i** Immunoblot analysis in empty vector overexpression (EV), pep2-nc-AC027045.3 overexpression (OV), pep2-nc-AC027045.3-Start codon mutants-overexpression (Mutant) AGS cell lines. **j** Cell growth viability of WT, pep1-nc-OLMALINC KO, pep1-nc-OLMALINC KO-WT ORF back-complemented (Rescue), pep1-nc-OLMALINC KO-start codon mutant ORF back-complemented (Mutant) AGS cells determined using the MTT assay at the indicated time points. Data are presented as mean ± SEM. *n* = 3. Two-way ANOVA; **P < 0.01, ns, no significance. **k** Clone formation assay of WT, pep1-nc-OLMALINC KO, pep1-nc-OLMALINC KO-WT ORF back-complemented (Rescue), pep1-nc-OLMALINC KO-Start codon mutant ORF back-complemented (Mutant) AGS cells. Data are presented as means ± SEM. *n* = 3. One-way ANOVA followed by Tukey test; **P < 0.01, ns, no significance. **l** Cell growth viability of WT, pep5-nc-TRHDE-AS1 KO, pep5-nc-TRHDE-AS1 KO-WT ORF rescued (Rescue) or pep5-nc-TRHDE-AS1 KO-start codon mutant ORF rescued (Mutant) AGS cells determined using the MTT assay at the indicated time points. Data are presented as means ± SEM. *n* = 3. Two-way ANOVA; **P  <  0.01, ns, no significance. **m** Clone formation assay of WT, pep5-nc-TRHDE-AS1 KO, pep5-nc-TRHDE-AS1 KO-WT ORF rescued (Rescue) or pep5-nc-TRHDE-AS1 KO-start codon mutant ORF rescued (Mutant) AGS cells. Data are presented as means ± SEM. *n* = 3. One-way ANOVA followed by Tukey test; **P  <  0.01, ns, no significance. **n** Cell growth viability of WT, pep-nc-ZNF436-AS1 KO, pep-nc-ZNF436-AS1 KO-WT ORF back-complemented (Rescue), pep-nc-ZNF436-AS1 KO-start codon mutant ORF back-complemented (Mutant) AGS cells determined using the MTT assay at the indicated time points. Data are presented as means ± SEM. *n* = 3. Two-way ANOVA; **P < 0.01, ns, no significance. **o** Clone formation assay of WT, pep-nc-ZNF436-AS1 KO, pep-nc-ZNF436-AS1 KO-WT ORF back-complemented (Rescue), pep-nc-ZNF436-AS1 KO-start codon mutant ORF back-complemented (Mutant) AGS cells. Data are presented as means ± SEM. *n* = 3. One-way ANOVA followed by Tukey test; **P < 0.01, ns, no significance. **p** Cell growth viability of empty vector (EV), pep2-nc-AC027045.3-overexpression (OV) or pep2-nc-AC027045.3 start codon mutant-overexpression (Mutant) AGS cells determined using the MTT assay at the indicated time points. Data are presented as means ± SEM. *n* = 3. Two-way ANOVA; **P  <  0.01, ns, no significance. **q** Clone formation assay of empty vector (EV), pep2-nc-AC027045.3-overexpression (OV) or pep2-nc-AC027045.3 start codon mutant-overexpression (Mutant) AGS cells. Data are presented as means ± SEM. *n* = 3. One-way ANOVA followed by Tukey test; **P  <  0.01, ns, no significance.

To further validate the CRISPR screening results, we selected 250 high-confidence novel peptide candidates (≥ 30 aa, CPAT score ≥ 0.6, top phenotype scores) for in-depth molecular characterization through various assays (Fig. 3a).

First, we tested the coding capacity of these candidates using peptide-SFB fusion constructs expressed in HEK-293T cells. Successful peptide expression was detected for 191 out of the 250 candidates (Supplementary information, Table S12). We selected 80 candidates with suitable qPCR primers from the top 100 scoring, along with some randomly selected on-hit candidates, to evaluate the translational efficiency of their host lncRNA transcripts via polysome profiling.^78^ Remarkably, 75 out of 80 lncRNAs were associated with actively translating high-molecular-weight polysomes, confirming their coding potential (Fig. 3b; Supplementary information, Table S13).

To examine endogenous peptide expression, we employed a CRISPR/Cas9-mediated knockin strategy^79^ to generate 27 cell lines with C-terminal Flag-only epitope tags fused to peptides (Supplementary information, Fig. S4a). All 27 tagged peptides were successfully detected at their expected molecular weights by western blot (Fig. 3c; Supplementary information, Table S14). Furthermore, immunofluorescence staining corroborated the expression of several peptides (Fig. 3d). These findings provided compelling evidence of their translation.

We further developed specific antibodies against 4 candidates with appropriate epitope (pep1-nc-OLMALINC, pep5-nc-TRHDE-AS1, pep-nc-ZNF436-AS1, pep2-nc-AC027045.3) for downstream studies. The abundance of these peptides varied between tumor and normal cells (Fig. 3e). The RNA levels of them also exhibited similar differences across cell lines (Supplementary information, Fig. S4b‒e), suggesting their potential roles in oncogenesis. For the 3 peptides that showed higher expression in tumor cells, we further generated peptide KO cell lines, along with cell lines with wild-type (WT) or start codon-mutated ORF re-expression to validate their role in tumor cell proliferation (Fig. 3f‒i). The pep1-nc-OLMALINC, pep5-nc-TRHDE-AS1 or pep-nc-ZNF436-AS1 KO cell lines showed a significant decrease in proliferation and clone formation. And the re-expression of the WT ORF, but not the start codon-mutant ORF, restored proliferation-related phenotypes (Fig. 3j‒o; Supplementary information, Fig. S4f, g). For pep2-nc-AC027045.3 that showed lower expression in tumor cells, we found that its overexpression inhibited cell proliferation (Fig. 3p, q). These results demonstrate that the observed functional impacts require translation of the specific peptides rather than effects of the host lncRNA transcripts.

Collectively, these multi-pronged validation experiments provide compelling evidence for the existence of these peptides and confirm the functional roles of four peptides as identified by the CRISPR screening.

### Functional prediction of peptides via structure-based interactome mapping

While the CRISPR screening and validation experiments provided strong evidence for the functional impacts of these peptides, characterizing the working mechanisms for each individual peptide by wet experiment is inefficient given their large numbers. To predict their potential functional mechanisms, we employed a structure-based proteome-wide interaction mapping approach.

**Fig. 4:**
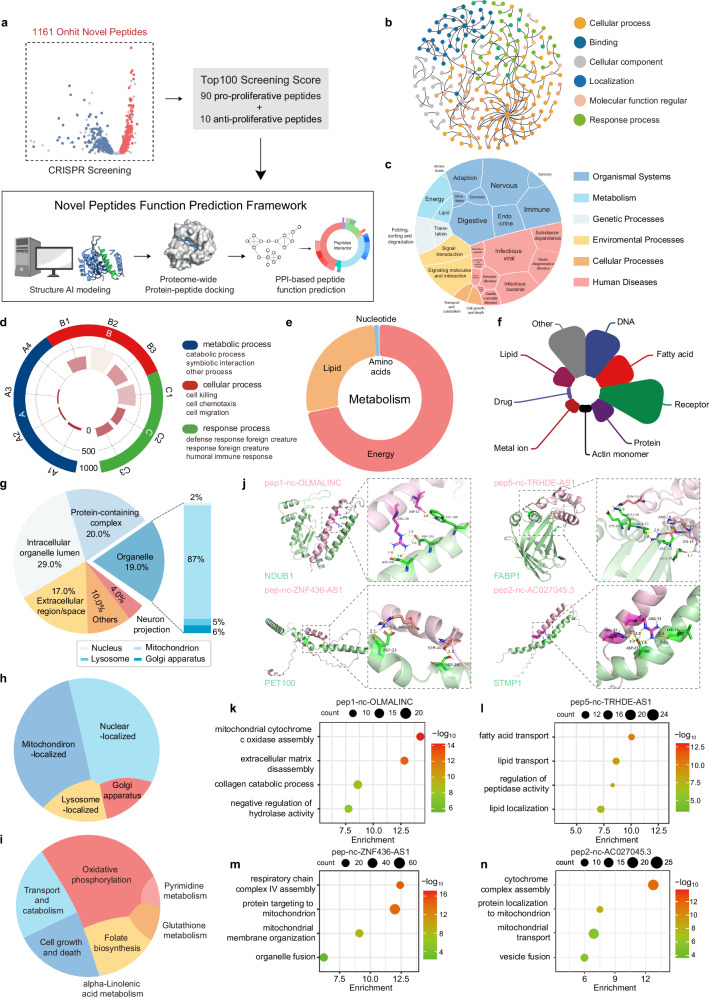
A framework for peptide function prediction based on AI structural prediction and peptide‒protein interaction networks. **a** Schematic diagram of the framework for peptide function prediction based on AI structural prediction and peptides‒protein interaction networks. **b** GO classification atlas for peptides based on predicted interaction protein. Candidates were assigned to six groups. The size of circles showed the degree of this node. **c** Comprehensive KEGG visualization using the Plotly-sunburst method. The KEGG classification is specific cellular processes, environmental information processing, genetic information processing, human diseases, metabolism, and organismal systems. **d** A circular histogram of representative process. Each histogram shows the distribution of GO enrichment. **e** A circular histogram of “Metabolism” process. Each histogram shows the distribution of KEGG enrichment. **f** Nightingale rose diagram visualization of the ‘binding’ function of each novel peptide. The color indicates the type of different combinations, and the radius of the arc indicates the number of related GO. **g** Pie bars show disaggregated information about the associated protein in each component of cell. The organelle of the pie chart is broken down into bars and further subdivided into nucleus, mitochondrion, lysosome, and Golgi apparatus. **h** Voronoi dendrogram visualization of organelle positions for peptides with specific localization. **i** Voronoi dendrogram visualization of the predicted interacting proteins of these peptides enriched by 6 specific KEGG items in Cellular Processes and Metabolism. **j** Visualization of pep1-nc-OLMALINC-NDUB1 (upper left), pep5-nc-TRHDE-AS1-FABP1 (upper right), pep-nc-ZNF436-AS1-PET100 (lower left) and pep2-nc-AC027045.3-STMP1 (lower right) binding by use of ClusPro molecular docking. Peptides in either complex are represented by lightpink “cartoon” structures, while protein receptors are represented by palegreen “cartoons”. The interaction residue label is displayed, and the specific measurement value of the polar contacts are represented in numerical form. **k** GO analyses of potential interacting protein of OLMALINC. **l** GO analyses of potential interacting protein of pep5-nc-TRHDE-AS1. **m** GO analyses of potential interacting protein of pep-nc-ZNF436-AS1. **n** GO analyses of potential interacting protein of pep2-nc-AC027045.3.

For the top 100 peptides with significant phenotypic effects identified from the CRISPR screening, comprising 90 pro-proliferative and 10 anti-proliferative peptides, we initially generated highly accurate structural models using AlphaFold2.^80^ Despite their short sequences, over 70% of these peptides attained a pLDDT score > 50 (Supplementary information, Fig. S5a and Table S15). These models were subsequently subjected to computational docking against the human proteome to identify their potential interacting proteins.^81,82^ To ensure the reliability of the docking results, we applied a threshold PRM score above 1.04 (compared to the regular value of 0.955)^81^ to refine the list of interacting proteins for each peptide. We then subsequently performed gene ontology (GO) enrichment analysis. To integrate the information regarding peptides, their interacting proteins, and associated GO terms, we employed a modular community segmentation algorithm. This facilitated the construction of a structure-based peptide‒protein interactome and functional network^83,84^ (Fig. 4a).

Upon classification and analysis of the GO terms linked to the majority of peptides in the network, we observed shared functional themes among these peptides. These peptides are involved in a variety of cellular biological activities and physiological processes^85^ (Fig. 4b, c; Supplementary information, Fig. S5b). Some peptides are closely associated with crucial biological processes such as the regulation of cellular metabolism, determination of cell fate, and stress responses (Fig. 4d; Supplementary information, Fig. S5c). A significant proportion of these peptides related to metabolic processes are specifically linked to energy metabolism (Fig. 4e), corroborating previous findings that many peptides are localized in mitochondria. Furthermore, it indicated that these peptides have the capacity to bind various types of molecules, including nucleic acids, lipids, and receptor proteins, thereby facilitating diverse functions and potentially participating in their transport processes (Fig. 4f; Supplementary information, Fig. S5d). Notably, the peptides frequently adopted scaffold-like conformations within cellular components, consistent with their generally short lengths (Fig. 4g). The peptides also exhibited diverse predicted subcellular localization patterns (Fig. 4h). Furthermore, the predicted interacting proteins of these peptides were involved in various metabolic processes such as oxidative phosphorylation, folate biosynthesis, and metabolite transport. This suggests that these peptides play a crucial role in cellular metabolism, corroborating the proliferation phenotypes observed using the CRISPR screening (Fig. 4i).

Focusing on the subset of peptides with successful validation (pep1-nc-OLMALINC, pep5-nc-TRHDE-AS1, pep-nc-ZNF436-AS1, pep2-nc-AC027045.3), the structure-based interactome analysis reinforced their positioning at key nodes within functional networks and associations with regulatory complexes (Supplementary information, Fig. S5e). Further detailed analysis was performed and these peptides were predicted to interact with organelle-localized proteins. Specifically, pep1-nc-OLMALINC, pep-nc-ZNF436-AS1 and pep2-nc-AC027045.3 were predicted to interact with mitochondrial protein NDUB1, PET100, and STMP1, respectively; pep5-nc-TRHDE-AS1 was predicted to interact with lysosomal protein FABP1 (Fig. 4j). Several interactions, including the representative predictions mentioned above, were further validated through Flag knockin peptide IP-MS analysis, thereby enhancing the credibility of this prediction framework (Supplementary information, Fig. S6a‒h and Table S16). Moreover, these peptides were predicted to be involved in organelle-related pathways: pep1-nc-OLMALINC was associated with the assembly of cytochrome c oxidase (Fig. 4k), pep5-nc-TRHDE-AS1 was implicated in fatty acid transport processes (Fig. 4l), pep-nc-ZNF436-AS1 was associated with the assembly of respiratory chain complex IV (Fig. 4m), and pep2-nc-AC027045.3 was associated with the assembly of cytochrome complex (Fig. 4n).

In summary, this integrative structure-based interactomics framework enabled systematic functional prediction for these peptides. The composite findings suggest their diverse roles as protein complex scaffolds and metabolic regulators. These insights pave the way for deeper mechanistic dissection of specific peptide functions and their pathogenic contributions.

### Functional validation of organelle-localized peptides that regulate metabolism processes

**Fig. 5:**
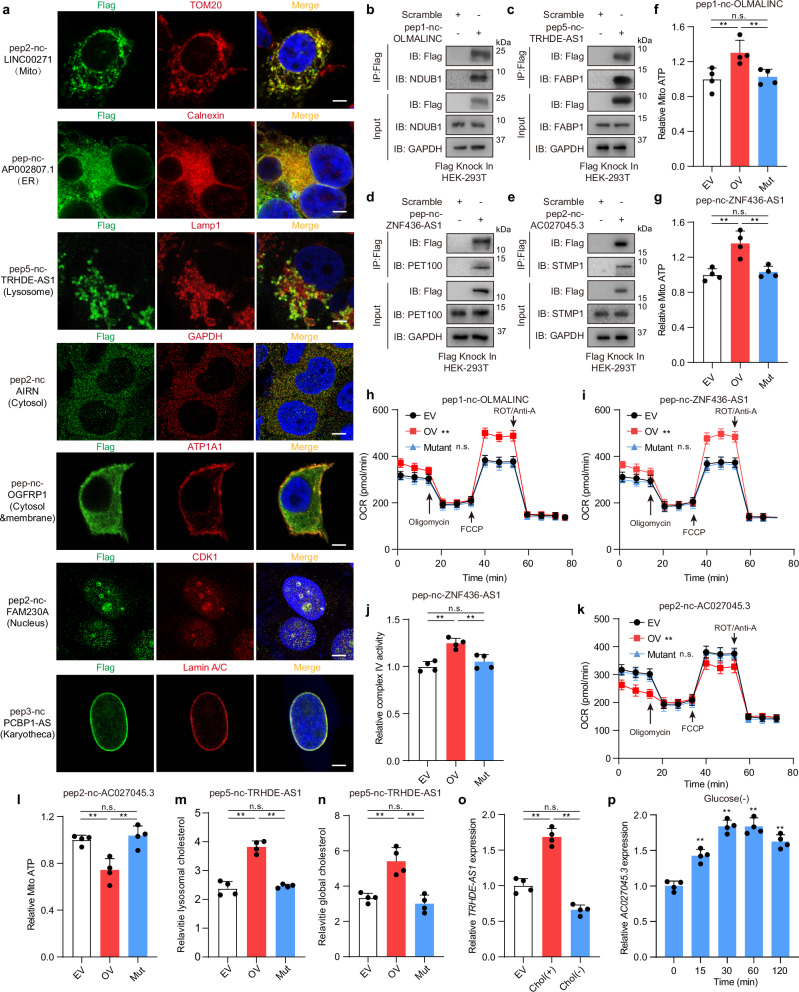
Novel peptide affects cellular metabolic processes by regulating organelle function. **a** Peptides (Flag) and specific cell organelles (mitochondria: TOM20; endoplasmic reticulum: Calnexin; lysosome: LAMP1; membrane: ATP1A1; cytosol: GAPDH; nucleus: CDK1; Karyotheca: Lamin A/C) in AGS cells were detected by immunofluorescence staining. Scale Bar: 10 μm. **b** Co-IP analysis of the interaction between pep1-nc-OLMALINC-Flag and NDUB1 in pep1-nc-OLMALINC-Flag knockin HEK-293T cell line. **c** Co-IP analysis of the interaction between pep5-nc-TRHDE-AS1-Flag and FABP1 in pep5-nc-TRHDE-AS1-Flag knockin HEK-293T cell line. **d** Co-IP analysis of the interaction between pep-nc-ZNF436-AS1-Flag and PET100 in pep-nc-ZNF436-AS1-Flag knockin HEK-293T cell line. **e** Co-IP analysis of the interaction between pep2-nc-AC027045.3-Flag and STMP1 in pep2-nc-AC027045.3-Flag knockin HEK-293T cell line. **f** Relative mitochondrial ATP production was detected. Empty vector overexpression (EV), pep1-nc-OLMALINC overexpression (OV), start codon mutant pep1-nc-OLMALINC overexpression (Mutant) AGS cells were treated with recording buffer (with 5 mM 2-DG and 5 mM pyruvate) to determine ATP generation during mitochondrial ATP synthesis. One-way ANOVA followed by Tukey test; **P < 0.01, ns, no significance. **g** Relative mitochondrial ATP production was detected. Empty vector overexpression (EV), pep-nc-ZNF436-AS1 overexpression (OV), start codon mutant pep-nc-ZNF436-AS1 overexpression (Mutant) AGS cells were treated with recording buffer (with 5 mM 2-DG and 5 mM pyruvate) to determine ATP generation under mitochondrial ATP synthesis. One-way ANOVA followed by Tukey test; **P < 0.01, ns, no significance. **h** OCR profile was monitored in empty vector overexpression (EV), pep-nc-ZNF436-AS1 overexpression (OV), start codon mutant pep-nc-ZNF436-AS1 overexpression (Mutant) AGS cells with a Seahorse XF24 analyzer. The metabolic inhibitors were injected at different time points as indicated. Two-way ANOVA; **P  <  0.01, ns, no significance. **i** OCR profile was monitored in empty vector overexpression (EV), pep1-nc-OLMALINC overexpression (OV), start codon mutant pep1-nc-OLMALINC overexpression (Mutant) AGS cells with a Seahorse XF24 analyzer. The metabolic inhibitors were injected at different time points as indicated. Two-way ANOVA; **P  <  0.01, ns, no significance. **j** Relative complex IV activity was detected in empty vector overexpression (EV), pep-nc-ZNF436-AS1 overexpression (OV), start codon mutant pep-nc-ZNF436-AS1 overexpression (Mutant) AGS cells. One-way ANOVA followed by Tukey test; **P < 0.01, ns, no significance. **k** OCR profile was monitored in empty vector overexpression (EV), pep2-nc-AC027045.3 overexpression (OV), start codon mutant pep2-nc-AC027045.3 overexpression (Mutant) AGS cells with a Seahorse XF24 analyzer. The metabolic inhibitors were injected at different time points as indicated. Two-way ANOVA; **P  <  0.01, ns, no significance. **l** Relative mitochondrial ATP production was detected. Empty vector overexpression (EV), pep2-nc-AC027045.3 overexpression (OV), start codon mutant pep2-nc-AC027045.3 overexpression (Mutant) AGS cells were treated with recording buffer (with 5 mM 2-DG and 5 mM pyruvate) to determine ATP generation under mitochondrial ATP synthesis. One-way ANOVA followed by Tukey test; **P < 0.01, ns, no significance. **m** Lysosomal cholesterol was detected in empty vector overexpression (EV), pep5-nc-TRHDE-AS1 overexpression (OV), start codon mutant pep5-nc-TRHDE-AS1 overexpression (Mutant) AGS cells. One-way ANOVA followed by Tukey test; **P < 0.01, ns, no significance. **n** Cellular cholesterol was detected in empty vector overexpression (EV), pep5-nc-TRHDE-AS1 overexpression (OV), start codon mutant pep5-nc-TRHDE-AS1 overexpression (Mutant) AGS cells. One-way ANOVA followed by Tukey test; **P < 0.01, ns, no significance. **o** Cellular *TRHDE-AS1* expression levels under cholesterol stimulation (chol^+^) (50 μM, 2 h) and cholesterol deficiency (chol^–^) (0.5% methyl-β-cyclodextrin, 3 h) were detected in AGS cells. One-way ANOVA followed by Tukey test; **P < 0.01, ns, no significance. **p** Cellular *AC027045.3* expression levels under glucose deficiency time gradient (0–120 min) treatment were detected in AGS cells. One-way ANOVA followed by Tukey test; **P < 0.01, ns, no significance.

The structure-based interactome suggested specific subcellular localizations for 34 of the top 100 peptide candidates (Supplementary information, Table S17). To validate these predictions, we generated C-terminal Flag fusion constructs and performed immunofluorescence staining. Remarkably, 26 out of the 34 peptides exhibited localization patterns consistent with the predictions, distributed across various organelles and subcellular structures including lysosomes, mitochondria, endoplasmic reticulum, nuclei, and plasma membranes (Fig. 5a; Supplementary information, Table S17). Consistent with the abovementioned predictions of their functions, the four candidates of focused interest also exhibited specific localizations. pep1-nc-OLMALINC, pep-nc-ZNF436-AS1, pep2-nc-AC027045.3 were localized to mitochondria and pep5-nc-TRHDE-AS1 was localized to lysosomes (Fig. 5a; Supplementary information, Fig. S6i).

Further, we sought to validate the possible functional mechanisms of these four peptides suggested by the interactome and functional network analysis. We validated key protein interactions predicted for these peptides using a peptide-Flag knockin cell line in vivo, along with purified recombinant peptides and proteins in vitro (Supplementary information, Fig. S6j): pep1-nc-OLMALINC with NDUB1 (Fig. 5b; Supplementary information, Fig. S6k), pep5-nc-TRHDE-AS1 with FABP1 (Fig. 5c; Supplementary information, Fig. S6l), pep-nc-ZNF436-AS1 with PET100 (Fig. 5d; Supplementary information, Fig. S6m), and pep2-nc-AC027045.3 with STMP1 (Fig. 5e; Supplementary information, Fig. S6n). Functional assays further corroborated the peptides’ roles in regulating their predicted pathways. pep1-nc-OLMALINC and pep-nc-ZNF436-AS1 promoted mitochondrial ATP production (Fig. 5f, g), oxygen consumption (Fig. 5h, i), and complex activities (Fig. 5j), while pep2-nc-AC027045.3 inhibited these processes (Fig. 5k, l). Notably, these metabolic phenotypes depended on the peptide rather than host lncRNA transcripts. For pep5-nc-TRHDE-AS1, its lysosomal localization and interaction with FABP1 enhanced cholesterol metabolism, manifested by increased lysosomal and cellular cholesterol levels in pep5-nc-TRHDE-AS1 overexpressing AGS cell lines (Fig. 5m, n). Furthermore, *TRHDE-AS1* expression levels increased upon cholesterol supplementation but decreased with cholesterol depletion (Fig. 5o), while *AC027045.3* showed time-dependent upregulation following glucose starvation (Fig. 5p). These regulatory dynamics suggest that they exhibited metabolite-responsive expression patterns.

The important functions exhibited by these peptides have intrigued us to understand where they fit into the evolutionary process. We examined cross-species sequence conservation of the peptide-coding genomic loci. These peptides are not conserved among vertebrates. For example, the C-terminus of pep5-nc-TRHDE-AS1 is absent in almost all species except *Homo sapiens*, while the N-terminus of pep1-nc-OLMALINC is fully present only in primates (Supplementary information, Fig. S7a‒d). Interestingly, these peptides are highly conserved in species closely related to *Homo sapiens* (Supplementary information, Fig. S7e‒h), indicating relatively recent origins.

In summary, through an integrated multi-pronged approach, we have characterized key regulatory roles and mechanisms for a subset of newly identified peptides in this work. These peptides impact fundamental metabolic processes by interacting with organelle-located protein partners and dynamically responding to metabolic cues. These findings demonstrate that certain peptides are important metabolic regulators with origins tied to recent anthropoid evolution.

### In vivo functional characterization and clinical relevance of peptides in gastric cancer

**Fig. 6:**
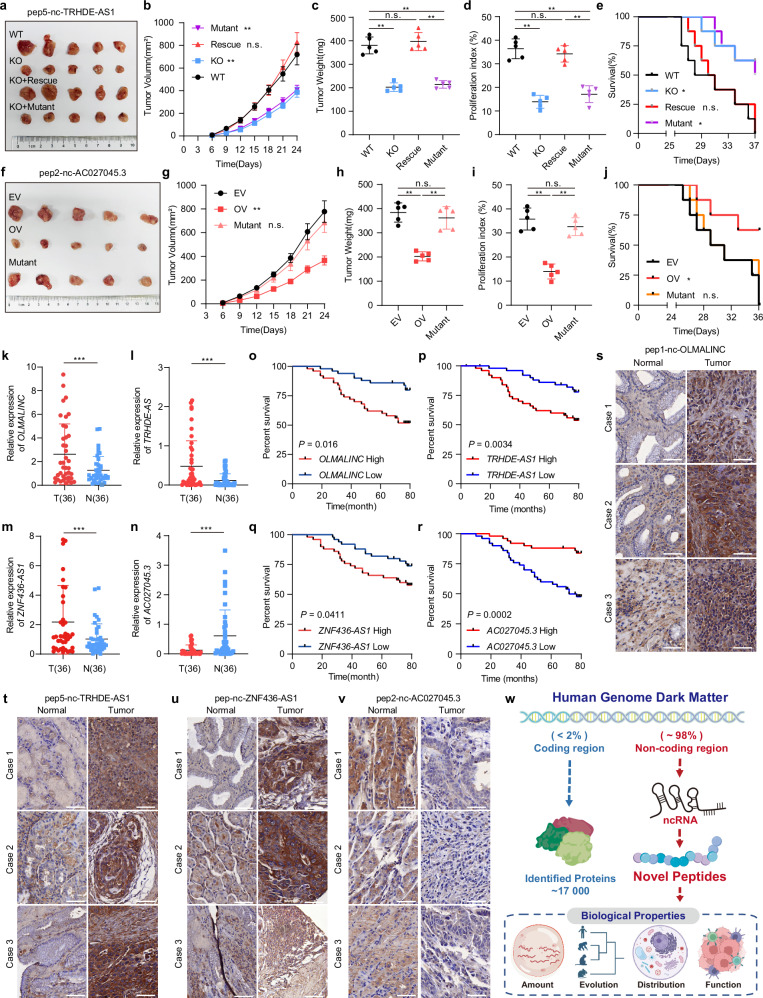
Clinical implications and therapeutic prospects of peptides in gastric cancer. **a** Xenograft mouse model established using WT, pep5-nc-TRHDE-AS1 KO, pep5-nc-TRHDE-AS1 KO-WT ORF back-complemented (Rescue) or pep5-nc-TRHDE-AS1 KO-start codon mutant ORF back-complemented (Mutant) AGS cells in BALB/c nude mice (*n* = 5 mice per group). In vivo generated tumors are depicted. **b** Analysis of WT, pep5-nc-TRHDE-AS1 KO, pep5-nc-TRHDE-AS1 KO-WT ORF back-complemented (Rescue) or pep5-nc-TRHDE-AS1 KO-start codon mutant ORF back-complemented (Mutant) AGS cell tumor growth and volume in the xenograft Balb/c nude mouse model. Data are presented as means ± SEM of *n*  =  5 mice per group. Two-way ANOVA; **P  <  0.01, ns, no significance. **c** Analysis of WT, pep5-nc-TRHDE-AS1 KO, pep5-nc-TRHDE-AS1 KO-WT ORF back-complemented (Rescue) or pep5-nc-TRHDE-AS1 KO-start codon mutant ORF back-complemented (Mutant) AGS cells tumor weight in the xenograft mouse model. Data are presented as means ± SEM of *n*  =  5 mice per group. One-way ANOVA followed by Tukey test; **P < 0.01, ns, no significance. **d** The relative intensities of Ki67 in indicated immunohistochemistry (IHC) staining of WT, pep5-nc-TRHDE-AS1 KO, pep5-nc-TRHDE-AS1 KO-WT ORF back-complemented (Rescue) or pep5-nc-TRHDE-AS1 KO-start codon mutant ORF back-complemented (Mutant) AGS cells were quantified by ImageJ. Data are presented as means values  ±  SD from *n* =  5 xenograft tumor samples per group. One-way ANOVA followed by Tukey test; **P < 0.01, ns, no significance. **e** Survival in the mice with WT, pep5-nc-TRHDE-AS1 KO, pep5-nc-TRHDE-AS1 KO-WT ORF back-complemented (Rescue) or pep5-nc-TRHDE-AS1 KO-start codon mutant ORF back-complemented (Mutant) AGS cells injected. *n* = 8 mice per group. Log-rank test; *P <  0.05, ns, no significance. **f** Xenograft mouse model established using empty vector (EV), pep2-nc-AC027045.3-overexpression (OV) or pep2-nc-AC027045.3 start codon mutant-overexpression (Mutant) AGS cells in BALB/c nude mice (*n* = 5 mice per group). In vivo generated tumors are depicted. **g** Analysis of empty vector (EV), pep2-nc-AC027045.3-overexpression (OV) or pep2-nc-AC027045.3 start codon mutant-overexpression (Mutant) AGS cell tumor growth and volume in the xenograft Balb/c nude mouse model. Data are presented as means ± SEM of *n*  =  5 mice per group. Two-way ANOVA; **P  <  0.01, ns, no significance. **h** Analysis of empty vector (EV), pep2-nc-AC027045.3-overexpression (OV) or pep2-nc-AC027045.3 start codon mutant-overexpression (Mutant) AGS cell tumor weight in the xenograft mouse model. Data are presented as means ± SEM of *n*  =  5 mice per group. One-way ANOVA followed by Tukey test; **P < 0.01, ns, no significance. **i** The relative intensities of Ki67 in indicated IHC staining of empty vector (EV), pep2-nc-AC027045.3-overexpression (OV) or pep2-nc-AC027045.3 start codon mutant-overexpression (Mutant) AGS cells were quantified by ImageJ. Data are presented as means ± SD from *n* =  5 xenograft tumor samples per group. One-way ANOVA followed by Tukey test; **P < 0.01, ns, no significance. **j** Survival in the mice with empty vector (EV), pep2-nc-AC027045.3-overexpression (OV) or pep2-nc-AC027045.3 start codon mutant-overexpression (Mutant) AGS cells injected. *n* = 8 mice per group. Log-rank test; *P <  0.05, ns, no significance. **k** The relative RNA levels of *OLMALINC* in the tumor are normalized to paired non-tumor tissue as differential expression values (T/N), Wilcoxon matched-pairs signed rank test; ***P <  0.001. **l** The relative RNA levels of *TRHDE-AS1* in the tumor are normalized to paired non-tumor tissue as differential expression values (T/N), Wilcoxon matched-pairs signed rank test; ***P <  0.001. **m** The relative RNA levels of *ZNF436-AS1* in the tumor are normalized to paired non-tumor tissue as differential expression values (T/N), Wilcoxon matched-pairs signed rank test; ***P <  0.001. **n** The relative RNA levels of *AC027045.3* in the tumor are normalized to paired non-tumor tissue as differential expression values (T/N), Wilcoxon matched-pairs signed rank test; ***P <  0.001. **o** Kaplan-Meier analysis of the overall survival curve for gastric cancer patients (Second Affiliated Hospital, School of Medicine Zhejiang University cohorts; *n* = 80) with low or high *OLMALINC* RNA level. The RNA levels were detected using qRT-PCR relative to *GAPDH*. Kaplan-Meier analysis along with the log-rank test. **p** Kaplan-Meier analysis of the overall survival curve for gastric cancer patients (Second Affiliated Hospital, School of Medicine Zhejiang University cohorts; *n* = 80) with low or high *TRHDE-AS1* RNA level. The RNA levels were detected using qRT-PCR relative to *GAPDH*. Kaplan-Meier analysis along with the log-rank test. **q** Kaplan-Meier analysis of the overall survival curve for gastric cancer patients (Second Affiliated Hospital, School of Medicine Zhejiang University cohorts; *n* = 80) with low or high *ZNF436-AS1* RNA level. The RNA levels were detected using qRT-PCR relative to *GAPDH*. Kaplan-Meier analysis along with the log-rank test. **r** Kaplan-Meier analysis of the overall survival curve for gastric cancer patients (Second Affiliated Hospital, School of Medicine Zhejiang University cohorts; *n* = 80) with low or high *AC027045.3* RNA level. The RNA levels were detected using qRT-PCR relative to *GAPDH*. Kaplan-Meier analysis along with the log-rank test. **s** The expressions of pep1-nc-OLMALINC in 72 primary human gastric cancer specimens (The Second Affiliated Hospital of Zhejiang University cohorts), detected using the IHC assay. Images of three representative cases (three tumor and paired non-tumor tissues) are shown. Scale bar: 100 µm. **t** The expressions of pep5-nc-TRHDE-AS1 in 72 primary human gastric cancer specimens (The Second Affiliated Hospital of Zhejiang University cohorts), detected using the IHC assay. Images of three representative cases (three tumor and paired non-tumor tissues) are shown. Scale bar: 100 µm. **u** The expressions of pep-nc-ZNF436-AS1 in 72 primary human gastric cancer specimens (The Second Affiliated Hospital of Zhejiang University cohorts), detected using the IHC assay. Images of three representative cases (three tumor and paired non-tumor tissues). Scale bar: 100 µm. **v** The expressions of pep2-nc-AC027045.3 in 72 primary human gastric cancer specimens (The Second Affiliated Hospital of Zhejiang University cohorts), detected using the IHC assay. Images of three representative cases (three tumor and paired non-tumor tissues) are shown. Scale bar: 100 µm. **w** Diagram of Novel Peptidomics.

After elucidating the cellular functions of these peptides, we investigated their pathophysiological importance using in vivo xenograft models focusing on the representative oncogenic pep5-nc-TRHDE-AS1 and tumor-suppressive pep2-nc-AC027045.3. Strikingly, KO of pep5-nc-TRHDE-AS1 or overexpression of pep2-nc-AC027045.3 potently inhibited tumor growth in BALB/c nude mice (Fig. 6a‒c, f‒h). Immunohistochemical analyses revealed a negative correlation between the levels of pep2-nc-AC027045.3 and the intensity of proliferation markers Ki67 and CD31, whereas the levels of pep5-nc-TRHDE-AS1 correlated positively with the intensity of these two markers (Fig. 6d, i; Supplementary information, Fig. S8a, b). Furthermore, mice with pep2-nc-AC027045.3 overexpression or pep5-nc-TRHDE-AS1 deficiency exhibited prolonged survival (Fig. 6e, j). Collectively, these in vivo data demonstrate pathophysiological importance of these peptides in regulating tumor malignancy.

To further assess their clinical relevance, we evaluated the expression of their host genes (*OLMALINC*, *TRHDE-AS1*, *ZNF436-AS1*, *AC027045.3*) in matched tumor/normal tissues from gastric cancer patients by RT-qPCR. Consistent with cellular phenotypes, the expression of *OLMALINC, TRHDE-AS1* and *ZNF436-AS1* was significantly upregulated in tumors (Fig. 6k‒m), while *AC027045.3* expressed higher in normal tissues (Fig. 6n). Stratifying patients by median expression levels, survival analysis revealed that high *OLMALINC*/*TRHDE-AS1*/*ZNF436-AS1* or low *AC027045.3* expression correlated with poorer prognosis (Fig. 6o‒r), in line with previous reports linking elevated *TRHDE-AS1* expression to poor gastric cancer outcomes.^86^

Immunohistochemistry confirmed higher peptide abundances of pep1-nc-OLMALINC, pep5-nc-TRHDE-AS1 and pep-nc-ZNF436-AS1 in tumors compared to matched normal samples, while pep2-nc-AC027045.3 exhibited the converse pattern (Fig. 6s‒v). These concordant RNA and protein level differences between malignant and non-malignant states further nominate these peptides as potential cancer biomarkers.

Further, we hypothesized that these endogenously produced peptides might serve as biocompatible agents suitable for drug development. We validated proliferative regulatory effects for two peptides, pep2-nc-SNHG14 and pep-nc-AL365361.1, on AGS cells through in vitro synthesis and administration, with suitable EC_50_/IC_50_ values for drug development (Supplementary information, Fig. S8c‒f).

In summary, through in vivo tumor models and clinical analyses, we demonstrate the profound physiological impacts of specific peptides in modulating cancer pathogenesis. These findings hold considerable promise for therapeutic peptide applications.

## Discussion

The discovery and functional annotation of novel peptides are pivotal frontiers in proteomics and genomics.^3,39^ Technological constraints have impeded the identification of the coding products of certain transcripts, particularly those of ncRNAs. In our study, the advent of an ultrafiltration-based tandem MS strategy circumvented the limitations imposed by conventional protein interference, achieving high-precision discovery of 8945 novel noncanonical peptides from human gastric cancer-related samples.

Our CRISPR screening has identified 1161 peptide candidates that influenced AGS cell proliferation. Notably, the majority of these peptides appear to function as pro-proliferative regulators, consistent with prior research,^48^ which align with the proliferative nature of cancer cells. However, it is important to recognize that potential false negatives due to discrepancies between gastric cancer tissues and AGS cell lines may lead to an underestimation of the functional peptides. Consequently, broadening the scope of screening conditions and models, including in vivo tumor metastasis assays, could unveil a more detailed functional landscape of these peptides.

Consistent with recent findings, the majority of the novel peptides are evolutionarily young,^50^ underscoring their evolutionary significance and potential roles in cellular function, especially in the complex assembly processes of higher organisms. Notably, a significant fraction of the novel peptides localize to mitochondria, as supported by our findings and previous studies.^17,21,27,47,87,88^ Their evolutionary attributes and mitochondrial localization may reflect the increased metabolic and proliferative demands of higher organisms. Further research is necessary to advance our understanding of the genetic and cellular properties of these peptides.

Our studies highlight a series of peptides with regulatory roles in gastric cancer cell proliferation, with a subset validated in animal models and clinical samples, underscoring their potential as biomarkers. Particularly, peptides expressed specifically in tumors tissues could be of diagnostic significance. However, the direct identification of these peptides by MS is challenging due to their low abundance and short lengths. Integrating transcriptomic, translatomic, and proteomic analyses may overcome these challenges.^38^

Furthermore, the endogenously produced short peptides with antitumor properties are promising candidates for therapeutic development. For instance, our study indicated that pep2-nc-SNHG14, an 18-aa peptide, emerges as a potential drug development candidate. Moreover, our MS assay identified more than 700 peptides with fewer than 20 aa that fulfill the criteria for short-sequence endogenous peptides with drug discovery potential. Comprehensive functional analyses of these peptides and subsequent refinements of peptides and delivery systems are required to pave the way for these peptides to become valuable pharmaceutical assets.

The field of “cryptic peptidomics” is expanding rapidly (Fig. 6w). However, previous studies have shown minimal overlap in identified peptides, suggesting a largely unexplored proteome. Our study has likely revealed only a fraction of the novel human peptidome. It is also noteworthy that 4097 peptides we identified are supported by a single PSM. The existence of these peptides identified by our MS analysis needs a more rigorous validation. Consequently, we have uploaded their annotated spectra to the public database for review by the scientific community (see Data Availability). While some of these peptides have been validated in our studies through functional proteomics or molecular biology approaches, a significant portion remains to be systematically validated. Additionally, this study has focused exclusively on gastric cancer, uncovering only a small portion of the novel peptidome. A significant majority of this peptidome still requires thorough investigation. This immense undertaking stresses the need for a collective effort to aggregate and integrate data from other novel peptide or proteomic research. In support of this, we established the Human Novel Peptides Atlas Database (http://hmpa.zju.edu.cn/), a regularly updated repository for novel peptide research. This aims to compile a complete dataset of novel peptides, providing a valuable resource for researchers and enhancing our understanding of the human peptidome.

### Contact for reagent and resource sharing

Further requests for information, resources and reagents should be directed to and will be replied by the corresponding authors, Aifu Lin (linaifu@zju.edu.cn) and Tianhua Zhou (tzhou@zju.edu.cn).

## Materials and methods

### Cell lines

The human embryonic kidney cell line HEK293T (RRID: CVCL_0063), the human gastric cancer cell line AGS (RRID: CVCL_0139), the human gastric cancer cell line MKN45 (RRID: CVCL_0434), the human papillomavirus-related endocervical adenocarcinoma cell line HeLa (RRID: CVCL_0030) and human gastric mucosal epithelial cells GES-1 (RRID: CVCL_EQ22) were purchased from the National Collection of Authenticated Cell Cultures (China). All cells were negatively tested for mycoplasma contamination and authenticated based on short tandem repeat fingerprinting before use.

### Mice

All animal experiments were performed by a protocol approved by the Institutional Animal Care and Use Committee. Care of experimental animals followed guidelines and was approved by the Laboratory Animal Committee of Zhejiang University. Female nude mice (Balb/c strain; 4–6 weeks old) were purchased from the Shanghai Laboratory Animals Center and used in the xenograft mouse model assay. Animals were housed in a pathogen-free barrier environment (around 20 °C with 40% humidity and 12-h dark/light cycle) throughout the study. Mice were fed a normal chow diet and water with ad libitum feeding.

### Tissue samples

Fresh gastric cancer and normal tissues for mircopeptide MS-detection were obtained from the Second Affiliated Hospital, School of Medicine, Zhejiang University. All samples were collected from patients with informed consent, and all related procedures were performed with the approval of the internal review and ethics boards of the Second Affiliated Hospital, School of Medicine, Zhejiang University.

Another 90 patients with complete clinicopathologic characteristics and follow-up data who underwent surgery at the Second Affiliated Hospital, School of Medicine, Zhejiang University, and were histologically diagnosed with GC, were enrolled. Histological cancer types were evaluated by two independent pathologists by the TNM staging guide (2016) released by The American Joint Committee on Cancer (AJCC). Tissue microarrays were made by paraffin-embedded consecutive sections. We performed IHC staining for 72 of 90 tissues with complete tissue form for the following analysis. All patients were provided with informed written consents for obtaining study specimens. Experiments were approved by the Ethics Committee of the Second Affiliated Hospital, School of Medicine Zhejiang University. Detailed clinical information is listed in Supplementary information, Table S19.

### Cloning procedures

The full-length sORF was cloned from HEK293T or AGS cDNA by PCR. All eukaryotic overexpression genes were cloned into a pcDNA3.1-Flag empty vector or PLVX-SFB empty vector using ClonExpress II One Step Cloning Kit (Vazyme).

### Putative sORF database (RLNPORF) construction

To construct the preliminary sORF database, we utilized the prepare-orfs tool from the Ribotricer software suite (https://github.com/smithlabcode/ribotricer). This tool facilitated the extraction of potential open reading frames (ORFs) from the raw fasta files and GTF annotation files. The construction process involved the following parameters: “--gtf” parameter was employed to import the reference gene annotation files, which can be downloaded from the Ensembl database (Hg38, v103, http://ftp.ensembl.org/pub/release-103/gtf/homo_sapiens/); ”--fasta” parameter was used to import the sequence files, also available for download from the Ensembl database (http://ftp.ensembl.org/pub/release-103/fasta/homo_sapiens/dna/); “--start_codons” parameter was set to recognize the four typical start codons (ATG/CTG/GTG/TTG) to ensure that the ORFs identified meet the criteria for initiating protein synthesis. Then the ORF sequences were extracted and jointed from the fasta file based on their genome coordinate. Finally, the amino acid sequences were generated by micropan (v2.1). The length of the translated amino acid sequences was calculated, and sequences longer than 250 aa were excluded from the final putative sORF database.

For the genetic and biological characterization annotation of these peptides, the location and classification information such as genome location, chromosome, transcript information, classification of transcript, start codon, etc. will be annotated by Ribotricer after ORF extraction and joint. Conservation score was calculated via Phastcons by default parameter, index of 20 mammalian data was downloaded from UCSC (https://hgdownload.cse.ucsc.edu/goldenPath/hg38/phastCons20way/). Coding probability score was calculated by CPAT (https://github.com/liguowang/cpat).

### LC-MS/MS detection and identification of sORFs

Snap frozen tissues were powdered using a pre-cooled mortar and pestle under the continuous addition of liquid nitrogen, on days with a humidity below 30%. 100 mg powdered tissue and appropriate amount of cells were resuspended in 2 mL lysis buffer (7 M Urea, 2 M Thiourea, 100 mM DTT, 4% CHAPS, 0.5 mM EDTA, 40 mM Tris, 2% NP40, 1% Triton X-100) and lysed by sonication. The supernatant was transferred to 30 kDa, 10 kDa and 3 kDa ultrafiltration tubes (Millipore; UFC9030, UFC9010, UFC 9003) and centrifuged at 13,000× *g* for 20 min. Protein lysates of 3‒10 kDa and < 3 kDa were used for subsequent MS analysis.

After precipitation, trypsin digestion (1:50 w/w), reduction (DTT, 5 mM), alkylation (IAM, 12 mM), and other treatments, the sample was then separated by a C18 column (75 μm inner-diameter, 360 μm outer-diameter, 150 mm length, 2 μm C18). Each sample has one injection. Bound peptides were then eluted over 70 min at a constant flow rate of 300 nL/min and the mobile phases water/0.1% FA and 80% ACN/0.1% FA (A and B, respectively) were applied in the effective linear gradients starting from 2% B and increasing to 28% in 58 min, followed by an increase to 35% B in 65 min, 98% B in 70 min. The Thermo Q Exactive HF-X mass spectrometer was programmed to operate in data-dependent mode using Xcalibur 4.1 software. The acquisition sequence began with a single full-scan mass spectrum in the Orbitrap (350–1800 m/z, 60,000 resolution), followed by 20 data-dependent MS/MS scans with a normalized collision energy of 30%. The AGC target was set as 3e6, and the maximum injection time was 50 ms. The MS^2^ spectra were acquired with 15,000 resolution. Each mass spectrum was analyzed using the Thermo Xcalibur Qual Browser and Proteome.

MS peptide sequences and, hence, protein identity was determined by matching fragmentation patterns in protein databases using the Mascot software program (Matrix Science, Boston, MA, USA). Enzyme specificity was set to partially tryptic with two missed cleavages. Modifications of the peptides included carboxyamidomethylation (cysteines, variable), oxidation (methionine, variable), phosphorylation (S, T, Y, H, variable) and acetylation (N-term, K, variable). Mass tolerance was set to 20 ppm for precursor ions and fragment ions. Spectral matches were filtered to make the false-discovery rate less than 1% at the peptide level using the target-decoy method. The proteins in UniprotKB_ Reviewed (Swiss-Prot) were set to ‘Contaminant’ to exclude their interference. The results of the spectral matches are annotated according to RLNPORF and manually filtered. The peptide whose ORF type is annotated as “annotated”, i.e., this is a protein/peptide that has been identified and included in UniProtKB_Reviewed database, is discarded (For the definition rules of ORF type, please refer to https://github.com/smithlabcode/ribotricer). IntORFs that are fully embedded within proteins included in the UniProtKB_Reviewed database are discarded. The search results from different samples will be compared, and we will check for redundant ORFs that overlap with each other. If redundant ORFs are identified and a longer ORF contains a unique sequence supported by a specific PSM, the shorter overlapping ORF will be discarded.

### Cell transfection, treatment, and lentiviral-based gene transduction

Human embryonic kidney cell line, HEK293T, human papillomavirus-related endocervical adenocarcinoma cell line, HeLa, human gastric mucosal epithelial cells, GES-1 and human gastric cancer cell line, MKN45 were maintained in DMEM supplemented with 10% FBS and human gastric cancer cell line, AGS, was maintained in F-12K supplemented with 10% FBS at 37 °C in 5% CO_2_ (vol/vol). All cells were negatively tested for mycoplasma contamination and authenticated based on short tandem repeat fingerprinting before use.

Lentiviral packaging vectors VSVG and psPAX2, along with an overexpression gene plasmid, were transfected into HEK-293T cells to produce lentiviruses. The virus was harvested 48 h and 72 h after transfection to transduce AGS cells, followed by selection with 3 µg/mL puromycin.

### RT-qPCR assay

TRIzol reagent (Invitrogen) was used to extract the associated RNAs according to the manufacturer’s instructions. Reverse transcription was performed using the iScript cDNA synthesis kit (Bio-Rad), and the abundance of target RNAs was detected by the iTaqTM Universal SYBR Green Supermix qPCR kit (Bio-Rad) according to the manufacturer’s instructions. Relative quantities of gene expression levels in each group were normalized to the reference genes (*Actin-β*, *GAPDH* or *U6*) and then normalized to the control group, as ${Exp}={2}^{{{\rm{\hbox{-}}}}\left(\left({{\rm{CT}}}\left({{\rm{target}}},{{\rm{test}}}\right){{\rm{\hbox{-}}}}{{\rm{CT}}}\left({{\rm{ref}}},{{\rm{test}}}\right)\right)-\left({{\rm{CT}}}\left({{\rm{target}}},{{\rm{control}}}\right){{\rm{\hbox{-}}}}{{\rm{CT}}}\left({{\rm{ref}}},{{\rm{control}}}\right)\right)\right)}$.

### Xenograft mouse model

All animal experiments were performed according to the protocol approved by the Institutional Animal Care and Use Committee. Mice were housed in a barrier facility proactive in environmental enrichment and fed with a normal chow diet and water ad libitum. Prepared tumor cells in 30 µL of sterile PBS were injected separately into the flanks of 4–6-week-old female BALB/c nude mice using the 100 μL sterile syringe. The tumor size was measured every 2 or 3 days using a calliper, and tumor volume was calculated using the standard formula: 0.54 × L × W^2^, where L refers to the longest diameter and W to the shortest diameter. Mice were euthanized when they met the institutional euthanasia criteria for the tumor size (length or width > 1.5 cm) or overall health condition. The solid tumors were removed, photographed, and weighed.

### Immunofluorescence

Cells were cultured in chamber slides overnight and fixed with 3.7% formaldehyde in PBS for 10 min at room temperature (RT), followed by permeabilization with 0.5% Triton X-100 in PBS for 10 min. Cells were then blocked with 5% FBS in PBS for 30 min at RT and incubated with the indicated primary antibody for 1 h at RT, followed by incubation with anti-rabbit (or mouse) IgG (H  +  L), F(ab′)2 fragment (Alexa Fluor 594 or 488 conjugate) from Abcam for 30 min at RT. Coverslips were mounted on slides using the anti-fade mounting medium with DAPI. Immunofluorescence (IF) images were acquired on an FV3000 confocal microscope (Olympus). For each channel, all images were acquired with the same settings. Fluorescence images were obtained using FV31S-SW Viewer (v2.3.1) and FV31S-DT (v2.3.1) software (Olympus).

### IHC staining

The paraffin-embedded tissues were deparaffinized in xylene followed by rehydration in a standard alcohol series, followed by antigen retrieval by 100 °C heating for 15 min in citrate buffer. The indicated primary antibody was diluted in 3% BSA and dropped to the tissue slides and incubated at 4 °C overnight. Slides were washed using PBS and incubated with 3% BSA diluted anti-rabbit or mouse HRP-secondary antibody for 60 min at RT. The slides were dehydrated in 50%, 70%, 80%, 95%, and 100% ethanol, and stabilized with a mounting medium. The images were acquired using an Olympus BX43 microscope with Olympus cellSens Dimension software. The quantification of IHC staining density was measured using ImageJ (Fiji v1.51j) software and calculated based on the average staining intensity and the percentage of positively stained cells.

### Cell lysis and immunoblotting

Cells were harvested in PBS and homogenized in NETN buffer (25 mM Tris-HCl (pH 8.0), 100 mM NaCl, 1 mM EDTA, and 0.5 mM dithiothreitol (DTT)) with protease inhibitor cocktail, phosphatase inhibitor cocktail, Panobinostat, and methylstat. Lysates were cleared by centrifugation at 13,000× *g* for 15 min at 4 °C. Supernatants were applied for immunoblotting (IB) with the indicated antibodies. The blotting signals were detected using Clarity Western ECL substrate (Bio-Rad). As for tagged-protein IP, the primary antibody and the protein A/G beads were replaced with Flag-M2 magnetic beads (Sigma), or HA magnetic beads (Pierce). The Flag-precipitated protein was eluted by 3× Flag Peptide (APExBio). Blot images were obtained using Image Lab v4.1 software (Bio-Rad).

### Polysome profiling

In total, 6 × 10^6^ Cells were treated with 100 μg/mL cycloheximide (CHX) (Sigma-Aldrich) for 5 min. Cells were lysed with polysome lysis buffer (15 mmol/L Tris-HCl, 5 mmol/L MgCl_2_, 100 mmol/L KCl, 2 mmol/L DTT, 1% Triton X-100, 100 μg/mL CHX). Cell lysates were centrifuged at 16,200× *g* at 4°C for 10 min. The supernatant was kept, and absorbance was measured at 260 nm. Then the supernatant was loaded onto the top of a 5% to 50% sucrose gradient (15 mmol/L Tris-HCl, 5 mmol/L MgCl_2_, 100 mmol/L KCl, 2 mmol/L DTT, 100 μg/mL CHX) and centrifuged at 210,000× *g* at 4 °C for 190 min (SW 41Ti ROTOR, Beckman). The gradient was collected into 12 fractions by monitoring RNA absorbance at 254 nm. RNA in each fraction was extracted and quantified by RT-qPCR. The enrichment of lncRNAs on polysome was normalized to the input sample.

### CRISPR/Cas9-based genome editing

For CRISPR/Cas9-based gene KO, designed gRNAs were inserted into Lenti-CRISPR v2 plasmid. Single-cell clones were selected, and PCR was performed using genomic DNA as a template.

For knockin, donor oligo was designed and commercially synthesized (Tsingke Biotech). The targeting vector was cotransfected with donor vector into cells, followed by selection with puromycin (3 μg/mL). The efficiency of knockin was examined by western blot and immunofluorescence to detect the expression of Flag.

For KO and back-complementation, the ORFs of peptides were cloned into pcDNA3.1 empty vector. Lentiviral packaging vectors VSVG and psPAX2, along with an overexpression gene plasmid, were transfected into HEK-293T cells to produce lentiviruses. The virus was harvested 48 h and 72 h after transfection for the transduction of sORF-KO AGS cells, followed by selection using 3 µg/mL puromycin. The efficiency of KO and back-complementation were detected by western blot.

The sgRNA information was listed in Supplementary information, Table S18.

### Conservativeness analysis and homologous sequence analysis

Primate and mammal alignment sequences of peptide-related genomic DNAs were downloaded from Ensembl database (https://ensembl.org/Homo_sapiens/Gene?compara_Alignments), evolutionary trees were generated by MEGA 11 (https://www.megasoftware.net/). Evolutionary trees and aligned sequences were visualized by ggtree (v3.4.4).

### Protein recombination and purification

Recombinant peptides MBP-pep-nc-ZNF436-AS1-His, MBP-pep1-nc-OLMALINC-His, MBP-pep5-nc-TRHDE-AS1-His and MBP-pep2-nc-AC027045.3-His were expressed in *E. coli* strain BL21-CodonPlus (DE3)-RIPL (Agilent Technologies) and purified using Ni-NTA Sefinose Resin (Sangon Biotech). Recombinant protein NDUB1-GST, FABP1-GST, STMP1-GST and PET100-GST were expressed in *Escherichia coli* strain BL21-CodonPlus (DE3)-RIPL (Agilent Technologies) and purified using GST magnetic beads (Sangon Biotech). The concentration and purity of recombinant proteins were measured by SDS-PAGE and Coomassie staining with the standard BSA control.

### In vitro protein pull-down assay

GST-tagged protein purified using GST magnetic beads was incubated with His-tagged protein (1–3 µg) purified with the NTR-Ni resin purified in 500 µL of binding buffer (50 mM Tris-HCl (pH 7.9), 10% glycerol, 100 mM KCl, 5 mM MgCl_2_, 10 mM β-mercaptoethanol and 0.1% NP-40) for 2 h at 4 °C with gentle rotation. Then, beads were washed with NETN buffer three times for 5 min each at 4 °C with rotation. Then, beads were eluted with 50 μL of 2× SDS loading buffer, and the eluted protein or protein complexes were detected by IB.

### Clinical feature analysis of sORF-related genes

Clinical features and expression data of gastric cancer in TCGA database were download from UCSC data center. Clinical associated groups were defined as follows: gastric cancer (GC: differentially expressed genes between all gastric expression data and all normal data); early gastric cancer (EGC: differentially expressed genes between all T1 stage gastric expression data and all normal data); Helicobacter pylori (HP: differentially expressed genes between positive helicobacter pylori data and negative data among tumor samples); metastasis (differentially expressed genes between M1 data and M0 data among tumor samples); recurrence (differentially expressed genes between recurrence data and non-recurrence data among tumor samples). Pathological classification: differentially expressed genes between sub-pathological expression data and normal data. Clinical stage classification: differentially expressed genes between clinical stage expression data and normal data. All differentially expressed genes from TCGA were analyzed by DESeq2 (v1.30.1). Dataset GSE122130 was used to analyze genes associated with cisplatin resistance. The expression data were downloaded from GEO database. Clean data were obtained after removing reads containing adapter and trimming low quality base with trim_galore (v0.0.1) (https://github.com/FelixKrueger/TrimGalore), reference genome was downloaded from Ensembl database. Index of the reference genome was built and paired-end clean reads were aligned to the reference genome using STAR (v2.7.4a). Differentially expressed genes between cisplatin-resistant and control group was analyzed by DESeq2 (v1.30.1). GSE128967 was a chemical therapy FOLFOX strategy-associated dataset with expression data downloaded from GEO database. Differentially expressed genes between primary resistance (PR) group or acquired drug (AR) resistance group and control group were analyzed by DESeq2 (v1.30.1). GSE154127 was 5FU-resistance-associated dataset, differentially expressed genes between 5FU-resistant and control group were analyzed by online tool geo2r from NCBI (default analyzed package: limma version). All significantly changed genes were defined as absolute value of log_2_tranformed fold change > 1 and adjusted *P* < 0.05. Copy number data of targeted genes were downloaded from UCSC data center, sORFs-related genes were labeled.

### CRISPR screening and data analysis

sgRNAs for the ORFs in this study were designed using the Broad Institute GPP sgRNA designer for *Streptococcus pyogenes* Cas9 against genome coordinates for the GRCh38 assembly of the human genome (https://portals.broadinstitute.org/gpp/public/analysis-tools/sgrna-design). Only the exon coding region of the ORF and its 5’UTR (defined as within 1000 bp of the start codon) were designed for sgRNAs, with up to eight sgRNAs designed for the targeting coding region and up to four sgRNAs for the 5’UTR using CRISPick (https://portals.broadinstitute.org/gppx/crispick/public). The library contains both 341 positive controls and 1041 untargeted sgRNA.

Optimal infection conditions were determined in each cell line to achieve 30%–50% infection efficiency, corresponding to a multiplicity of infection (MOI) of ~0.5–1. Spin infections were performed in 6 cm^2^ dish with 3 × 10^7^ cells to achieve a representation of at least 1000 cells per sgRNA following puromycin selection. Approximately 24 h after infection, all wells within a replicate were pooled and were split into T225 flasks. 24 h after infection, cells were selected with puromycin for 7 days to remove uninfected cells. After selection was complete, 2.0 × 10^7^ cells were harvested for assessment of the initial abundance of the library. Cells were passaged every 3–4 day and harvested ~21 day after infection. 2.0 × 10^7^ cells were harvested for assessment of the final abundance of the library. The whole genome of the cells was extracted and then subjected to PCR and sequencing. Read counts were normalized to reads per million and then log_2_ transformed. For analysis, the phenotype score for each peptide was designated as the average log_2_fold change in the reads of sgRNAs targeting its coding region after 10 doublings, which were analyzed using DESeq2. Higher phenotype scores suggest a stronger inhibition of cell proliferation, while lower scores imply a promotion of cell proliferation. Cutoff values are set at 1 and ‒1, with scores greater than 1 indicating a significant inhibitory effect and scores less than ‒1 indicating a significant promotional effect on cell proliferation. Based on these scores and the specified cutoff values, sORFs exhibiting minimal effects on the 5ʹ UTR ( | 5ʹUTR Phenotype score | < 1) and strong effects on the coding regions ( | Coding region Phenotype score | > 1) were filtered and categorized as on-hit-sORFs.

### Molecular docking

The 3D structures of peptides and proteins (e.g., FABP1 and TRHDE-AS1) were retrieved from AlphaFold DB. In docking analysis, the peptides are considered as ligands while proteins are considered as receptors. Calculations were performed in the ClusPro 2.0 web server following its default rigid body docking guidelines. We run simulations without constraints, leaving the ligands free to search the entire protein surface for the most favorable binding sites. The software PyMol (version 2.5) was used to examine conformational models of potential hydrogen bonds.

### Functional enrichment analysis of peptides

All structures of peptides were obtained by AlphaFold2 with top5 prediction models and we used a deep attention model for the identification of peptide binding sites, PepNN. The main potential interacting proteins, identified by applying a PRM score threshold greater than 1.04 (typically set at 0.955 in other studies), were subjected to GO analysis. The cellular component, molecular functions and biological processes were annotated using the GO database (G.O.; http://www.geneontology.org) and those GO terms were mapped to GO hierarchy for their parents and ancestors by GOATOOLS Python library. The peptides atlas visualization was used based on the Modularity community segmentation algorithm by GEPHI tool.

## Supplementary information

Fig. S1

Fig. S2

Fig. S3

Fig. S4

Fig. S5

Fig. S6

Fig. S7

Fig. S8

Table S1

Table S2

Table S3

Table S4

Table S5

Table S6

Table S7

Table S8

Table S9

Table S10

Table S11

Table S12

Table S13

Table S14

Table S15

Table S16

Table S17

Table S18

Table S19

## Data Availability

The fasta file (RLNPORF and RLNPORF_decoy) and the annotated spectra of the peptides with single PSM in this study (ms_psm1) are available for download at Figeshare (https://figshare.com/articles/journal_contribution/Reference_Library_of_Novel_Peptide_ORFs/26831161). The raw MS files were deposited in ProteomeXchange (mRNA-derived pepteides: PXD041392, Username: reviewer_pxd041392@ebi.ac.uk, Password: kC2ycIYM, ncRNA-derived peptides: PXD041397, Username: reviewer_pxd041397@ebi.ac.uk, Password: 2KnjbP7l).

## Code availability

The codes of RLNPORF construction is available for download at github (https://github.com/suxww/RLNPORF-Construction.git).
