# Publisher Correction: Comprehensive discovery and functional characterization of the noncanonical proteome

**DOI:** 10.1038/s41422-025-01091-x

**Published:** 2025-03-10

**Authors:** Chengyu Shi, Fangzhou Liu, Xinwan Su, Zuozhen Yang, Ying Wang, Shanshan Xie, Shaofang Xie, Qiang Sun, Yu Chen, Lingjie Sang, Manman Tan, Linyu Zhu, Kai Lei, Junhong Li, Jiecheng Yang, Zerui Gao, Meng Yu, Xinyi Wang, Junfeng Wang, Jing Chen, Wei Zhuo, Zhaoyuan Fang, Jian Liu, Qingfeng Yan, Dante Neculai, Qiming Sun, Jianzhong Shao, Weiqiang Lin, Wei Liu, Jian Chen, Liangjing Wang, Yang Liu, Xu Li, Tianhua Zhou, Aifu Lin

**Affiliations:** 1 The Center for RNA Medicine, International Institutes of Medicine, International School of Medicine, The 4th Affiliated Hospital of Zhejiang University School of Medicine, Yiwu, Zhejiang China; 2 MOE Laboratory of Biosystem Homeostasis and Protection, College of Life Sciences, Zhejiang University, Hangzhou, Zhejiang China; 3 Cancer Center, Zhejiang University, Hangzhou, Zhejiang China; 4 Key Laboratory of Cancer Prevention and Intervention, China National Ministry of Education, Hangzhou, Zhejiang China; 5 Department of Cell Biology and Program in Molecular Cell Biology, Zhejiang University School of Medicine, Hangzhou, Zhejiang China; 6 Department of Gastroenterology, the Second Affiliated Hospital, School of Medicine and Institute of Gastroenterology, Zhejiang University, Hangzhou, Zhejiang China; 7 Key Laboratory of Structural Biology of Zhejiang Province, Westlake Laboratory of Life Sciences and Biomedicine, Westlake University, Hangzhou, Zhejiang China; 8 Department of Gastrointestinal Surgery, The Second Affiliated Hospital, Zhejiang University School of Medicine, Hangzhou, Zhejiang China; 9 Zhejiang University-University of Edinburgh Institute, Zhejiang University School of Medicine, Haining, Zhejiang China; 10 The Second Affiliated Hospital, Zhejiang University School of Medicine, Hangzhou, Zhejiang China; 11 Hangzhou Cancer Hospital, Hangzhou, Zhejiang China; 12 Department of Nephrology, Center for Regeneration and Aging Medicine, The Fourth Affiliated Hospital of School of Medicine and International School of Medicine, International Institutes of Medicine, Zhejiang University, Yiwu, Zhejiang China; 13 Institute of Immunology, Zhejiang University School of Medicine, Hangzhou, Zhejiang China; 14 Department of Molecular Genetics, University of Toronto, Toronto, ON Canada; 15 Future Health Laboratory, Innovation Center of Yangtze River Delta, Zhejiang University, Jiashan, Zhejiang China; 16 Key Laboratory for Cell and Gene Engineering of Zhejiang Province, Hangzhou, Zhejiang China

**Keywords:** Molecular biology, Cell biology, Cancer

Correction to: *Cell Research* 10.1038/s41422-024-01059-3, published online 10 January 2025

The wrong Supplementary files (Table S12 and Table S13) were originally published with this article due to publisher’s mistake in correction stage; They have now been replaced with the correct files.

The original article has been corrected.

## Supplementary information

Table S12

Table S13

