# Supplementary material for: Comprehensive discovery and functional characterization of the noncanonical proteome
Source: Cell Res. 2025 Jan 10;35(3):186–204. doi: 10.1038/s41422-024-01059-3 (PMC11909191; doi:10.1038/s41422-024-01059-3)
Supplement: Supplementary file 1 — Fig. S1 [file 41422_2024_1059_MOESM1_ESM.pdf]

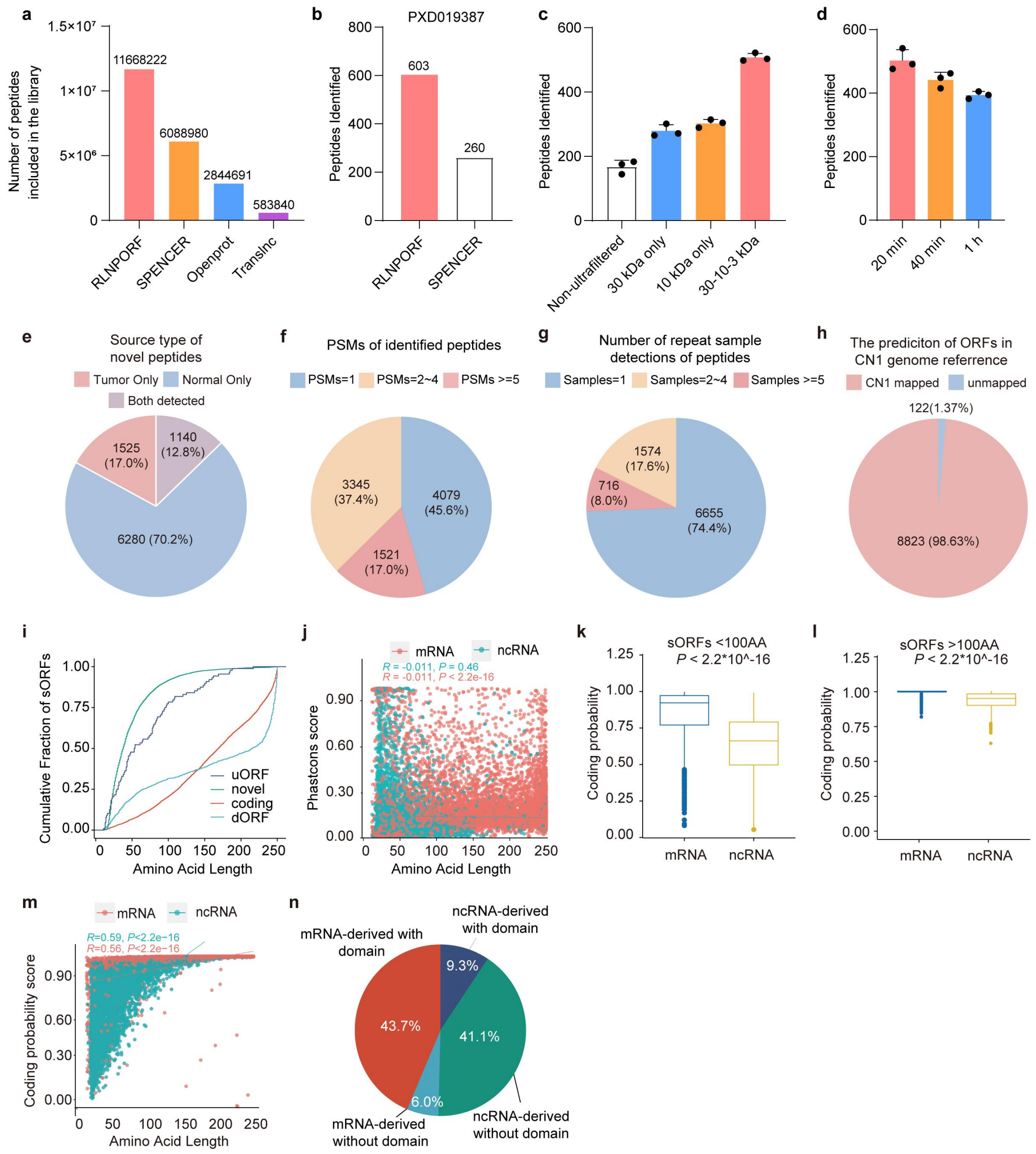


# Supplementary information, Figure S1

**(a)** Number of peptides included in RLNPORF and the ORF reference libraries of SPENCER, Openprot, and Translnc. **(b)** Number of peptides identified by spectrum matching of MS raw data in PDX019387 (gastric cancer) using ORF reference library of SPENCER with RLNPORF. **(c)** The number of peptides identified in AGS cells under different ultrafiltration conditions and the total ultrafiltration time was all set to 1 hour. **(d)** The number of peptides identified in AGS cells under the same 30-10-3

kDa gradient ultrafiltration conditions for different ultrafiltration times in each ultrafiltration tube (Total time: 1h, 2h and 3h). **(e)** The quantitative relationship between identified peptides and sample type from which they detected. **(f)** The quantitative relationship of PSMs of the identified peptides. **(g)** The quantitative relationship of peptides detected in different clinical samples/cell lines. **(h)** Matching results of the identified peptides with transcriptome data of CN1 genome reference. **(i)** Cumulative distribution of AA length peptides/protein from different sources. uORF: upstream ORF, dORF: downstream ORF, coding: classical protein, novel: peptides we identified. **(j)** Correlation between conservation score analyzed by Phastcons and AA length of peptides derived from ncRNA and mRNA. The correlations were characterized by the spearsman correlation coefficient. Each dot represents an individual novel peptide. **(k)** Boxplot for coding probability score comparison of peptides <100 AAs derived from mRNA and ncRNA analyzed by CPAT. Data are presented as individual value and statistic analysis was performed by Two-sided unpaired two-samples non-parametric Wilcoxon test, $p < 2.2e-16$. **(l)** Boxplot for coding probability score comparison of peptides ＞100 AAs and < 250 AAs derived from mRNA and ncRNA analyzed by CPAT. Data are presented as individual value and statistic by Two-sided unpaired two-samples non-parametric Wilcoxon test, $p < 2.2e-16$. **(m)** Correlation between coding probability score analyzed by CPAT and AA length of peptides derived from mRNA or ncRNA. The correlations were characterized by the spearsman correlation coefficient. **(n)** Pie map of domain mapping of peptides predicted by InterProScan.
