# Supplementary material for: Comprehensive discovery and functional characterization of the noncanonical proteome
Source: Cell Res. 2025 Jan 10;35(3):186–204. doi: 10.1038/s41422-024-01059-3 (PMC11909191; doi:10.1038/s41422-024-01059-3)
Supplement: Supplementary file 2 — Fig. S2 [file 41422_2024_1059_MOESM2_ESM.pdf]

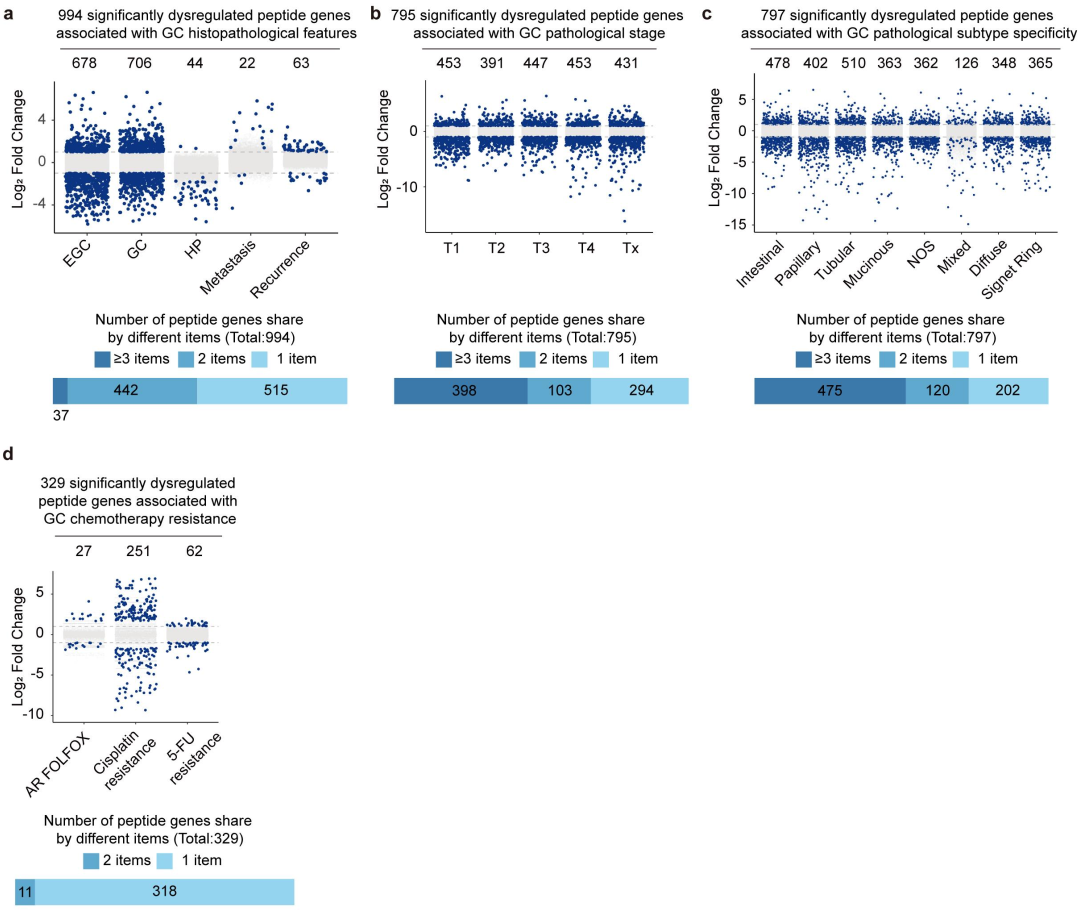

Supplementary information, Figure S2

(a) Upper:Manhattan map of the peptides genes related to histopathological features of gastric cancer, including early gastric cancer (EGC), gastric cancer (GC), helicobacter pylori infection (HP), Metastasis, Recurrence. Cutoff threshold of significantly changed genes was defined as $|log_2$ fold change$|>1$ and adjusted p value <0.05. Data were presented as individual log_2 fold change value. Lower: The number of peptide genes shared by different items. **(b)** Upper:Manhattan map of peptides genes of which expression is gastric cancer stage-specific. Cutoff threshold of significant changed genes was defined as $|log_2$ fold change$|>1$ and adjusted p value <0.05, normal data from STAD in TCGA database was selected as control. Data were presented as individual log_2 fold change value. Lower: The number of peptide genes

shared by different items. **(c)** Upper:Manhattan map of peptides genes of which expression is gastric cancer pathological classification-specific. Peptides belonging to the genes that were significantlychanged among different gastric cancer pathogenic. Cutoff threshold of significant changed genes was defined as $|\log_2$ fold change$|>1$ and adjusted p value <0.05, normal data from STAD in TCGA database was selected as control. Data were presented as individual $\log_2$ fold change value. Lower: The number of peptide genes shared by different items. **(d)** Upper:Manhattan map of peptides genes related to chemotherapy-resistant, including cisplatin resistant, 5-FU resistant, and acquired FOLFOX resistant. Cutoff threshold of significant changed genes was defined as $|\log_2$ fold change$|>1$ and adjusted p value <0.05. Data were presented as individual $\log_2$ fold change value. Lower: The number of peptide genes shared by different items.
