# Supplementary material for: Comprehensive discovery and functional characterization of the noncanonical proteome
Source: Cell Res. 2025 Jan 10;35(3):186–204. doi: 10.1038/s41422-024-01059-3 (PMC11909191; doi:10.1038/s41422-024-01059-3)
Supplement: Supplementary file 3 — Fig. S3 [file 41422_2024_1059_MOESM3_ESM.pdf]

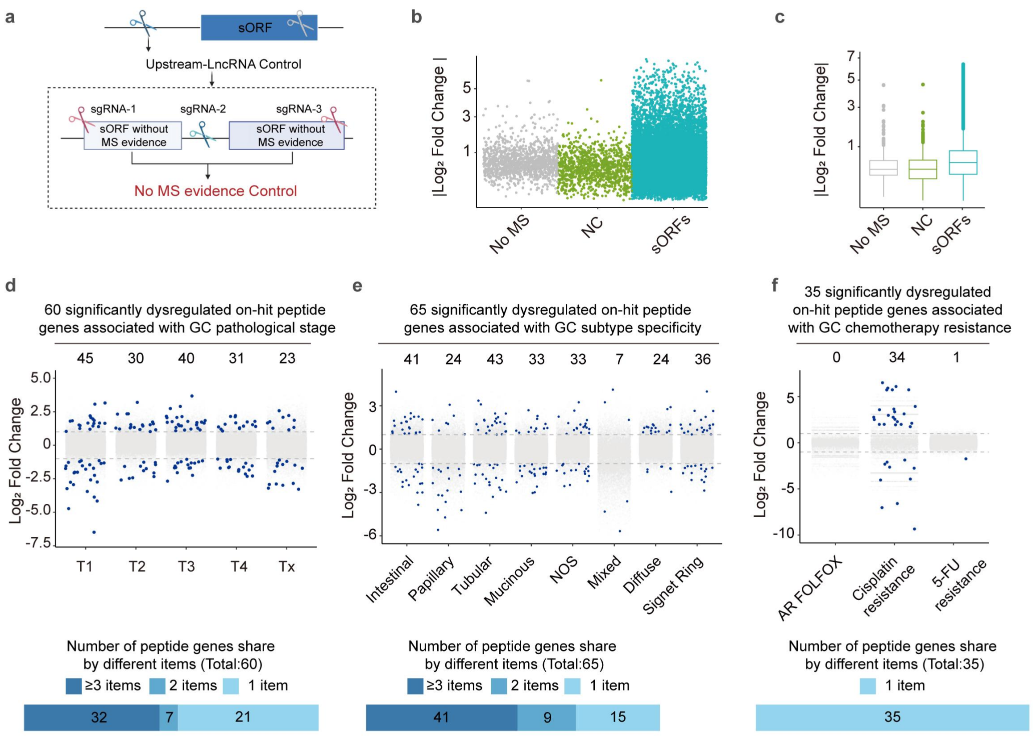

Supplementary information, Figure S3

(a) Schematic design of CRISPR screening for control group without MS evidence. Jitter **(b)** and box **(c)** plots show the absolute values of fold changes for sgRNAs, grouped by no MS evidence control (No MS), negative control (NC), and sORFs. **(d)** Upper: Manhattan plot of screening peptides from genes with expression specific to gastric cancer stages. The threshold for significantly changed gene was set at $|\log_2$ fold change$|>1$ and an adjusted p-value <0.05, using normal data from the STAD in the TCGA database as control. Data were presented as individual $\log_2$ fold change values. Lower: The number of peptide genes shared by different items. **(e)** Upper: Manhattan plot of screening peptides from genes with expression specific to gastric cancer pathological classifications. The threshold for significantly changed gene was set at $|\log_2$ fold change$|>1$ and an adjusted p-value <0.05, using normal data from the STAD in the TCGA database as control. Data were presented as individual $\log_2$ fold

change values. Lower: The number of peptide genes shared by different items. **(f)** Upper: Manhattan plot of screening peptides related to chemotherapy-resistance, including resistance to cisplatin, 5-FU, and acquired FOLFOX. The threshold for significantly changed gene was set at $|\log_2$ fold change$|>1$ and an adjusted p-value <0.05. Data were presented as individual $\log_2$ fold change values. Lower: The number of peptide genes shared by different items.
