# Supplementary material for: Comprehensive discovery and functional characterization of the noncanonical proteome
Source: Cell Res. 2025 Jan 10;35(3):186–204. doi: 10.1038/s41422-024-01059-3 (PMC11909191; doi:10.1038/s41422-024-01059-3)
Supplement: Supplementary file 4 — Fig. S4 [file 41422_2024_1059_MOESM4_ESM.pdf]

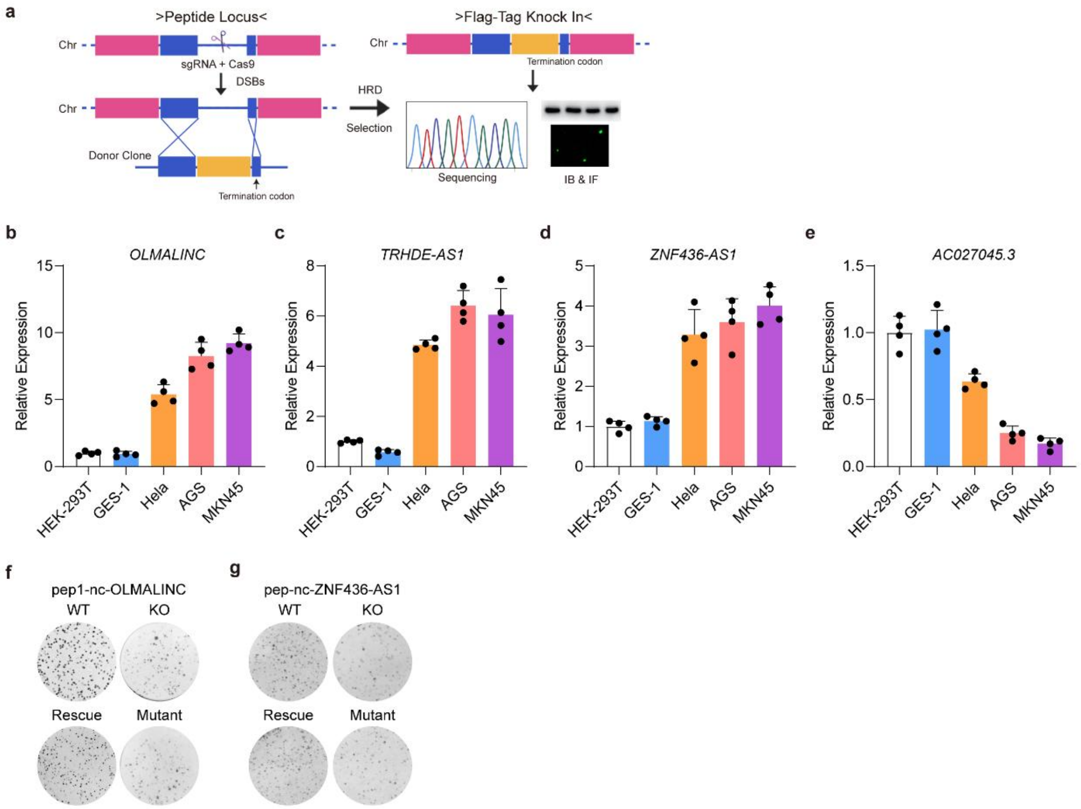

Supplementary information, Figure S4

(a) Schematic map of the CRISPR Knock-In process. **(b)** RNA expression of *OLMALINC* in HEK-293T, GES-1, HeLa, AGS, and MKN45 cells. Data were presented as mean ± SEM. **(c)** RNA expression of *TRHDE-AS1* in HEK-293T, GES-1, HeLa, AGS, and MKN45 cells. Data were presented as mean ± SEM. **(d)** RNA expression of *ZNF436-AS1* in HEK-293T, GES-1, HeLa, AGS, and MKN45 cells. Data were presented as mean ± SEM. **(e)** RNA expression of *AC027045.3* in HEK-293T, GES-1, HeLa, AGS, and MKN45 cells. Data were presented as mean ± SEM. **(f)** Clone formation assay of wildtype (WT), pep1-nc-OLMALINC knockout (KO), pep1-nc-OLMALINC knockout with wildtype ORF back-complemented (Rescue), and pep1-nc-OLMALINC knockout with start codon mutant ORF back-complemented (Mutant) AGS cells. **(g)** Clone formation assay of wildtype (WT),

pep-nc-ZNF436-AS1 knockout (KO), pep-nc-ZNF436-AS1 knockout with wildtype ORF back-complemented (Rescue), and pep-nc-ZNF436-AS1 knockout with start codon mutant ORF back-complemented (Mutant) AGS cells.
