# Supplementary material for: Comprehensive discovery and functional characterization of the noncanonical proteome
Source: Cell Res. 2025 Jan 10;35(3):186–204. doi: 10.1038/s41422-024-01059-3 (PMC11909191; doi:10.1038/s41422-024-01059-3)
Supplement: Supplementary file 5 — Fig. S5 [file 41422_2024_1059_MOESM5_ESM.pdf]

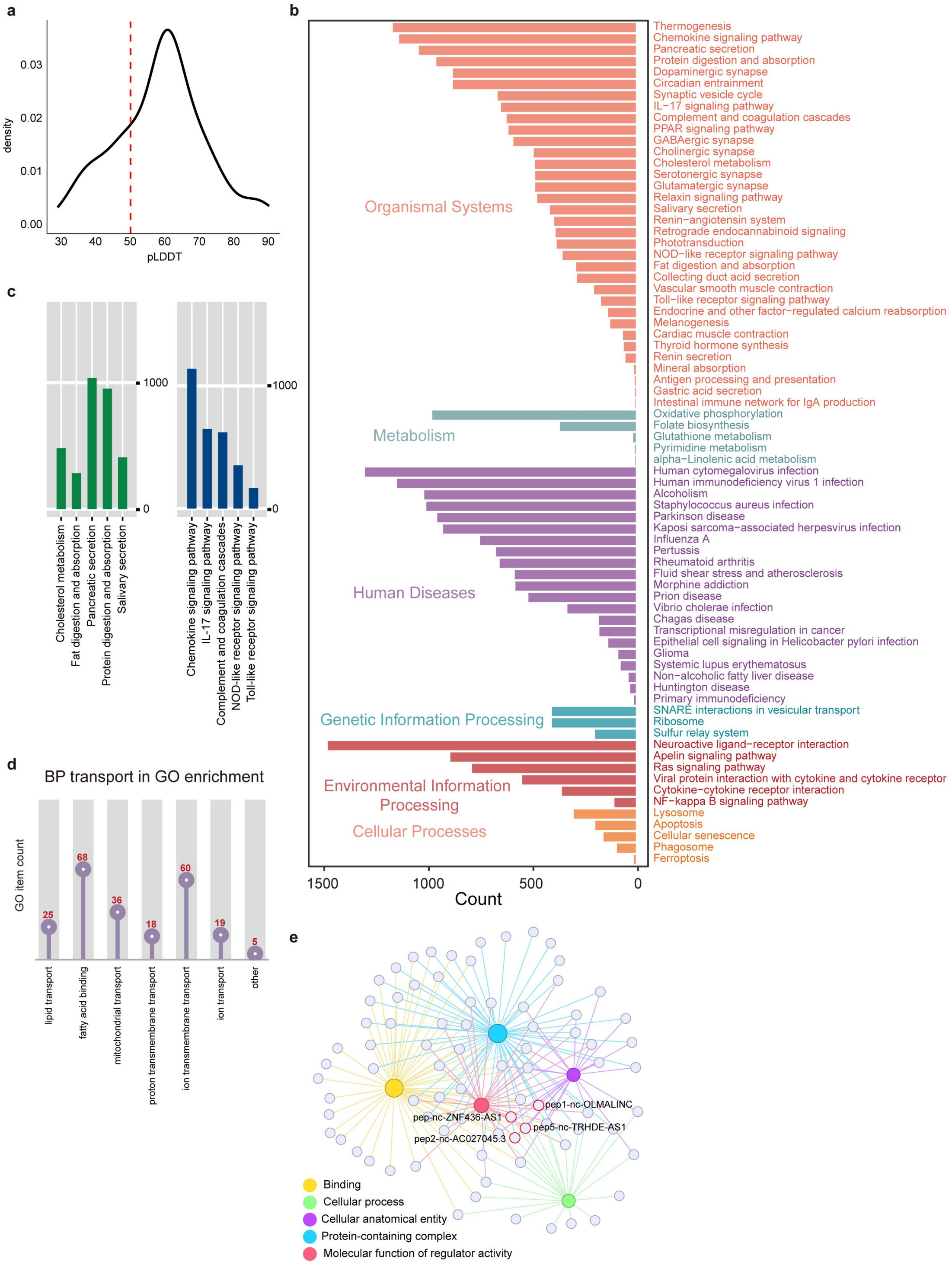

Supplementary information, Figure S5

(a) The density plot of each peptide's pLDDT score is shown. The Y-axis represents the density of results from AlphaFold2, visualized as the frequency or distribution of

specific pLDDT score ranges. The X-axis displays the pLDDT scores, which measure local distance differences and indicate the accuracy of the predicted peptide structure. A red dashed line, set at a confidence threshold of 50, divides the density distribution into regions of confidence. **(b)** Histogram presentation of KEGG classification. Bars represent the number of matches for each KEGG term across six categories: cellular processes (orange), environmental information processing (red), genetic information processing (blue), human diseases (purple), metabolism (gray), and organismal systems (pink). **(c)** Bar chart of enriched KEGG pathways in the "Metabolism" and "Signaling Pathway" classes. The vertical axis represents pathway names, and the horizontal axis represents gene counts. Green: Metabolism class; Blue: Signaling Pathways class. **(d)** The stem bar with background shows the enrichment results related to the GO term "transport" of different substances. **(e)** A Gephi network visualizes the 100 peptides and demonstrates the number of shared functions among the 5 GO enrichment terms. pep-nc-ZNF436-AS1, pep1-nc-OLMALINC, pep5-nc-TRHDE-AS1, and pep-nc-ZNF436-AS1 are labeled.
