# Supplementary material for: Comprehensive discovery and functional characterization of the noncanonical proteome
Source: Cell Res. 2025 Jan 10;35(3):186–204. doi: 10.1038/s41422-024-01059-3 (PMC11909191; doi:10.1038/s41422-024-01059-3)
Supplement: Supplementary file 6 — Fig. S6 [file 41422_2024_1059_MOESM6_ESM.pdf]

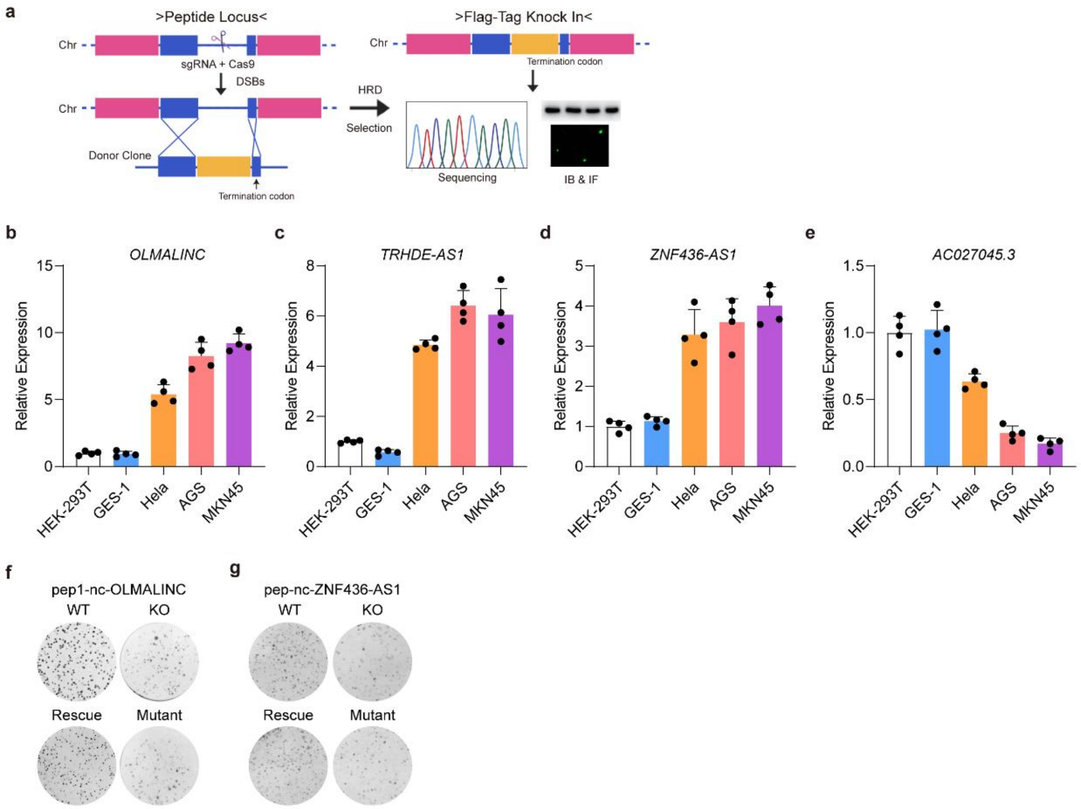

Supplementary information, Figure S6

(a) Representative interacting proteins of pep1-nc-OLMALINC identified by IP-MS. **(b)** Venn diagram comparing interacting proteins of pep1-nc-OLMALINC identified by IP-MS and predicted by the Alphafold2-PepNN model; Pearson's chi-squared test, $P < 0.0001$. **(c)** Representative interacting proteins of pep5-nc-TRHDE-AS1 identified by IP-MS. **(d)** Venn diagram comparing interacting proteins of pep5-nc-TRHDE-AS1 identified by IP-MS and predicted by the Alphafold2-PepNN model; Pearson's chi-squared test, $P < 0.0001$. **(e)** Representative interacting proteins of pep-nc-ZNF436-AS1 identified by IP-MS. **(f)** Venn diagram comparing interacting proteins of pep-nc-ZNF436-AS1 identified by IP-MS and predicted by the Alphafold2-PepNN model; Pearson's chi-squared test, $P < 0.0001$. **(g)** Representative interacting proteins of pep2-nc-AC027045.3 identified by IP-MS. **(h)** Venn diagram

comparing interacting proteins of pep2-nc-AC027045.3 identified by IP-MS and predicted by the Alphafold2-PepNN model; Pearson's chi-squared test, $P < 0.0001$.

(i) Coomassie-stained gel showing purified NDUB1-GST, FABP1-GST, STMP1-GST, PET100-GST, MBP-pep-nc-ZNF436-AS1-His, MBP-pep1-nc-OLMALINC-His, MBP-pep5-nc-TRHDE-AS1-His, and MBP-pep2-nc-AC027045.3-His. **(j)** His pull-down assay: Recombinant NDUB1-GST and MBP-pep1-nc-OLMALINC-His were purified, and the interaction between NDUB1 and pep1-nc-OLMALINC was detected by immunoblot assay. **(k)** His pull-down assay: Recombinant FABP1-GST and MBP-pep5-nc-TRHDE-AS1-His were purified, and the interaction between FABP1 and pep5-nc-TRHDE-AS1 was detected by immunoblot assay. **(l)** His pull-down assay: Recombinant PET100-GST and MBP-pep-nc-ZNF436-AS1-His were purified, and the interaction between PET100 and pep-nc-ZNF436-AS1 was detected by immunoblot assay. **(m)** His pull-down assay: Recombinant STMP1-GST and MBP-pep2-nc-AC027045.3-His were purified, and the interaction between STMP1 and pep2-nc-AC027045.3 was detected by immunoblot assay.
