# Supplementary material for: Comprehensive discovery and functional characterization of the noncanonical proteome
Source: Cell Res. 2025 Jan 10;35(3):186–204. doi: 10.1038/s41422-024-01059-3 (PMC11909191; doi:10.1038/s41422-024-01059-3)
Supplement: Supplementary file 7 — Fig. S7 [file 41422_2024_1059_MOESM7_ESM.pdf]

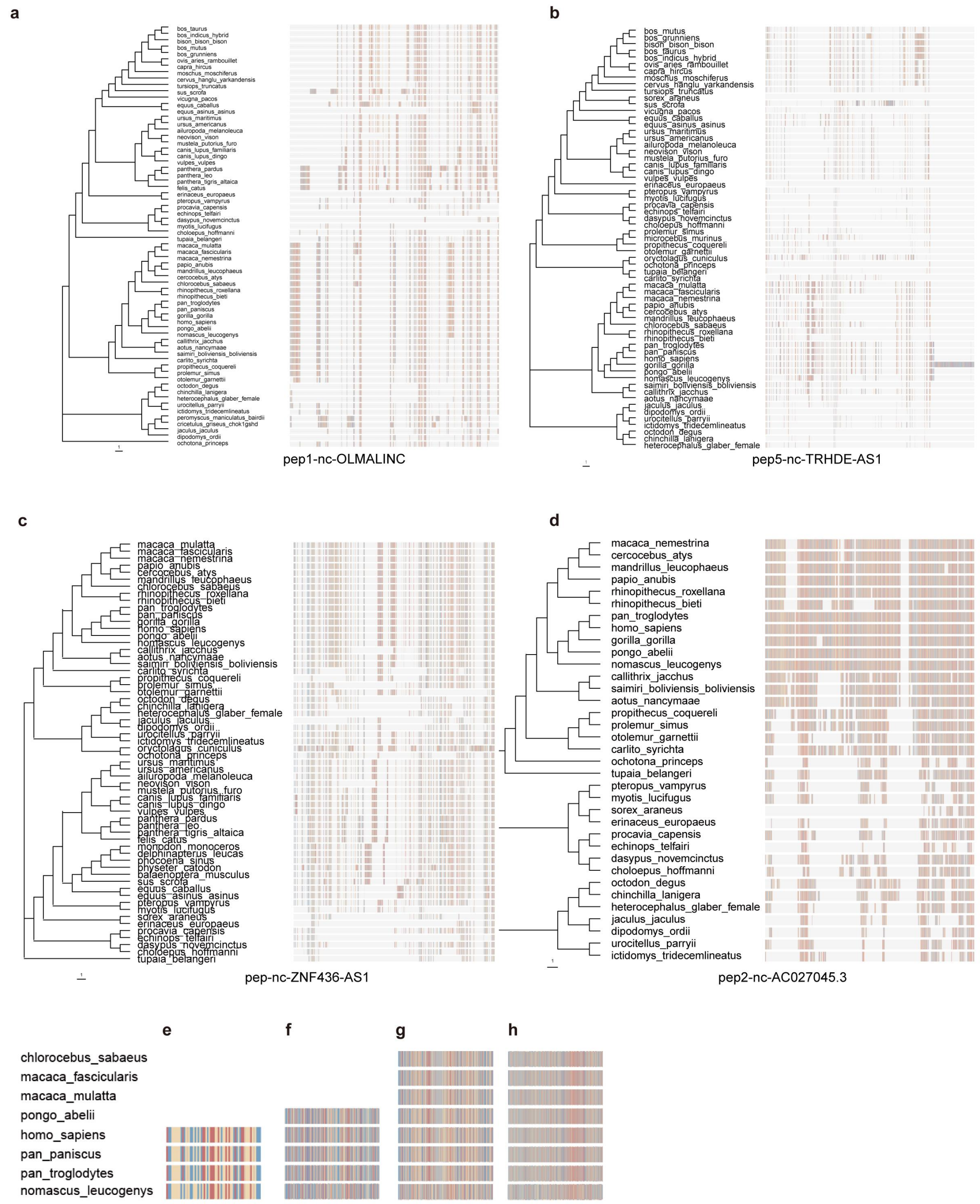

Supplementary information, Figure S7

(a) Conservation analysis of pep1-nc-OLMALINC among mammals. **(b)** Conservation analysis of pep5-nc-TRHDE-AS1 among mammals. **(c)** Conservation analysis of pep-nc-ZNF436-AS1 among mammals. **(d)** Conservation analysis of pep2-nc-AC027045.3 among mammals. **(e-h)** Conservation analysis of pep2-nc-AC027045.3 **(e)**, pep1-nc-OLMALINC **(f)**, pep-nc-ZNF436-AS1 **(g)**,

pep5-nc-TRHDE-AS1 **(h)** among primates.
