# Supplementary material for: Comprehensive discovery and functional characterization of the noncanonical proteome
Source: Cell Res. 2025 Jan 10;35(3):186–204. doi: 10.1038/s41422-024-01059-3 (PMC11909191; doi:10.1038/s41422-024-01059-3)
Supplement: Supplementary file 8 — Fig. S8 [file 41422_2024_1059_MOESM8_ESM.pdf]

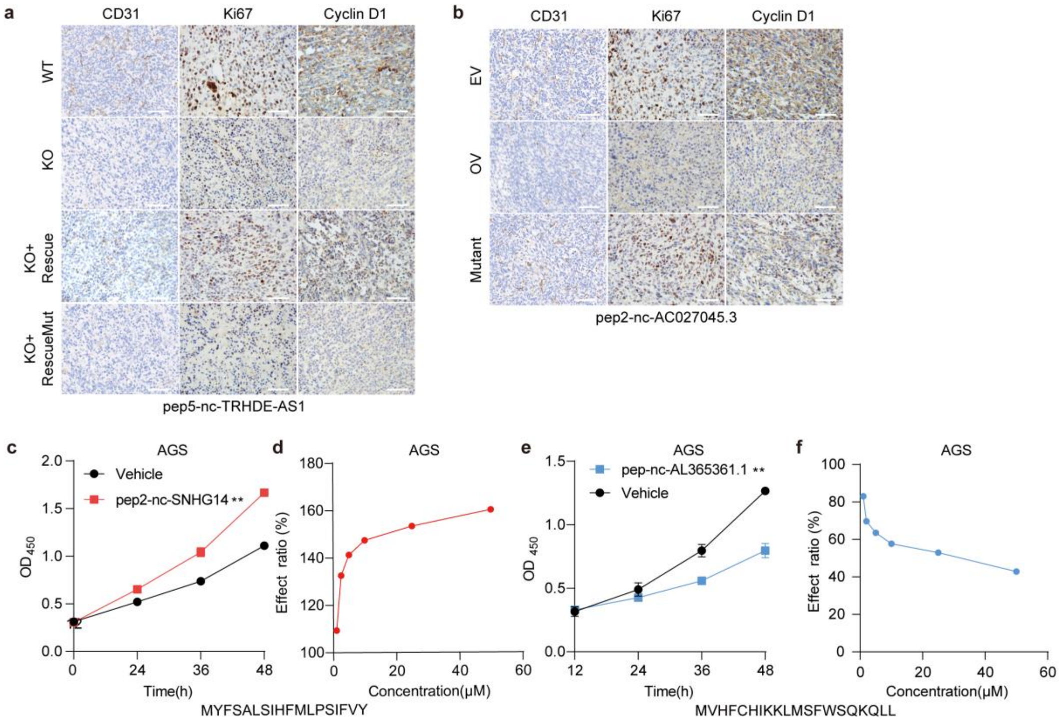

Supplementary information, Figure S8

(a) Representative IHC staining in randomly selected tumors from mice subcutaneously injected with the indicated pep5-nc-TRHDE-AS1-manipulated AGS cells. Scale bar: 100 µm. (b) Representative IHC staining in randomly selected tumors from mice subcutaneously injected with the indicated pep2-nc-AC027045.3-manipulated AGS cells. Scale bar: 100 µm. **(c)** Cell growth viability of AGS cells treated with 10 µM pep2-nc-SNHG14 for 12 hours or vehicle, determined using the MTT assay at the indicated time points. Data are presented as mean ± SEM, n=3. Two-way ANOVA; $^{**}P<0.01$. **(d)** Relationship between cell proliferation capacity and pep2-nc-SNHG14 concentration for 12-hour treatment, with a half-effect concentration of 31.4 µM. **(e)** Cell growth viability of AGS cells treated with 10 µM pep-nc-AL365361.1 for 12 hours or vehicle, determined using the MTT assay at the indicated time points. Data are presented as mean ± SEM, n=3.

Two-way ANOVA; ***P*<0.01. **(f)** Relationship between cell proliferation capacity and pep-nc-AL365361.1 concentration for 12-hour treatment, with a half-effect concentration of 35.6 μM.
