# Supplementary material for: Comprehensive discovery and functional characterization of the noncanonical proteome
Source: Cell Res. 2025 Jan 10;35(3):186–204. doi: 10.1038/s41422-024-01059-3 (PMC11909191; doi:10.1038/s41422-024-01059-3)
Supplement: Supplementary file 9 — Table S1 [file 41422_2024_1059_MOESM9_ESM.pdf]

Supplementary Information, Table S1. Basic and MS information for each sample

This table presents the basic and MS information for each sample in this study. It includes sample type, sample IDs, sample weight, the number of peptide identified by MS, the number of PSMs identified by MS, the number of positive decoy identified by MS.

Sample Type	Sample ID	Weight	The number of identified peptides	The number of identified PSMs	The number of positive decoys
Tumor Tissue	1	0.9	1481	5326	54
	2	0.43	267	1027	8
	3	0.51	377	1379	12
	4	0.31	106	398	2
	5	0.62	114	531	3
	6	0.37	113	360	2
Adjacent non-cancerous tissue	1	1.85	3135	15335	147
	2	0.98	1773	8871	77
	3	0.54	556	2249	22
	4	1.25	1738	8313	83
	5	1.34	1165	4921	49
	6	0.51	125	473	4
Normal gastric tissue & Cell	1	0.40	301	2420	24
	2	1.13	887	5088	50
	3	0.52	386	4804	49
	4	1.07	1237	6953	70
	5	1.34	1556	9907	101
	AGS	0.52	725	2388	18
