# Supplementary material for: Comprehensive discovery and functional characterization of the noncanonical proteome
Source: Cell Res. 2025 Jan 10;35(3):186–204. doi: 10.1038/s41422-024-01059-3 (PMC11909191; doi:10.1038/s41422-024-01059-3)
Supplement: Supplementary file 23 — Table S15 [file 41422_2024_1059_MOESM23_ESM.pdf]

Supplementary Information, Table S15. The pLDDT score for those peptides whose structures were predicted by Alphafold2

The table presents the pLDDT score (Scores corresponding to the most credible simulation results) for those peptide whose structure was predicted by Alphafold2.

Pep_name	ORF_id	pLDDT
pep1-nc-AL133313.1	ENST00000663643_68693906_68694295_390	28.8
pep-nc-SIGLEC10-AS1	ENST00000532688_51416044_51417326_492	32.2
pep2-nc-FAM230A	ENST00000624459_18492327_18492986_660	34.6
pep1-nc-AL359636.2	ENST00000431442_122607079_122607426_348	34.8
pep2-nc-GNG12-AS1	ENST00000413628_68202493_68202972_480	36.2
pep2-nc-LINC00271	ENST00000664629_135498552_135498815_264	36.6
pep3-nc-PCBP1-AS1	ENST00000442326_70094079_70102898_471	36.8
pep-nc-AC138028.5	ENST00000566114_88741672_88742195_345	36.8
pep-nc-MAPT-IT1	ENST00000624111_45897063_45897386_324	39.5
pep1-nc-LINC02175	ENST00000571219_25068280_25068537_258	39.7
pep-nc-AP002807.1	ENST00000526897_68052329_68053448_309	39.9
pep1-nc-AC006504.5	ENST00000585917_27797856_27806018_735	41.2
pep1-nc-BLACE	ENST00000378120_155358179_155358613_435	41.7
pep1-nc-AC006213.1	ENST00000586860_43999143_44002809_276	42.8
pep-nc-UMODL1-AS1	ENST00000329015_42103213_42103401_189	44.2
pep2-nc-AL159996.1	ENST00000674154_86757111_86757329_219	44.6
pep1-nc-AC127496.3	ENST00000576032_80940472_80941938_345	45.1
pep-nc-LINC02183	ENST00000637822_54545356_54546113_222	45.9
pep1-nc-AC135012.3	ENST00000599841_86193296_86193550_255	46.5
pep1-nc-OGFRP1	ENST00000332965_42277406_42277705_300	47.1
pep-nc-AC009053.3	ENST00000566506_74422164_74434472_309	47.7
pep-alt-LIMS2	ENST00000409754_127642192_127644103_249	48.5
pep2-nc-MAPT-AS1	ENST00000649665_45841832_45842092_261	48.7
pep-nc-AL355337.1	ENST00000659367_142679394_142679615_222	48.8
pep1-nc-LINC01344	ENST00000653755_182120585_182120830_246	49.6
pep-nc-ZNF436-AS1	ENST00000335648_23370095_23370370_276	50.7
pep2-nc-LAMTOR5-AS1	ENST00000626572_110355974_110418311_123	50.8
pep2-nc-AC010300.1	ENST00000600671_23325794_23326129_336	51.1
pep1-nc-AC099786.3	ENST00000565735_75122663_75122764_102	51.2
pep-nc-AC026412.4	ENST00000674095_1640665_1640865_201	52.2
pep1-nc-SPACA6P-AS	ENST00000602324_51685848_51686162_315	52.8
pep-nc-KCNQ1-AS1	ENST00000440887_2858784_2858891_108	52.9
pep-nc-AC009053.2	ENST00000563701_74367740_74367943_204	53.4
pep1-nc-LINC01570	ENST00000561572_5609996_5610154_159	53.6
pep-nc-AC068418.2	ENST00000577309_21458414_21458776_363	56.2
pep-nc-LINC02482	ENST00000667300_6658375_6658683_309	56.3
pep2-nc-AP001442.1	ENST00000600590_25941871_25942029_159	57.2

pep-nc-CTBP1-AS	ENST00000625256_1210407_1212117_303	57.2
pep-u-PSME1	ENST00000559123_24136177_24136302_126	57.4
pep1-nc-LINC00910	ENST00000592094_43377569_43377658_90	57.8
pep1-nc-AC018866.1	ENST00000421820_120709369_120710510_357	58.1
pep-nc-AC046158.1	ENST00000563408_78496193_78496351_159	58.2
pep1-nc-RN7SL200P	ENST00000585129_34685373_34685600_228	58.7
pep-nc-AC116428.1	ENST00000655943_109186486_109186668_183	58.7
pep2-nc-AL136964.1	ENST00000622038_107872411_107872536_126	58.8
pep2-nc-CCDC144NL-AS1	ENST00000577537_20902531_20902659_129	58.9
pep1-nc-AL021368.2	ENST00000606125_57911808_57911888_81	58.9
pep5-nc-TRHDE-AS1	ENST00000667465_72251783_72251968_186	58.9
pep1-nc-AC078881.1	ENST00000668108_177599309_177599515_207	59.5
pep-nc-AC018680.1	ENST00000500324_136395165_136395470_306	59.8
pep1-nc-LINC02269	ENST00000660497_173702172_173702261_90	59.9
pep1-nc-LINC01745	ENST00000430921_232718750_232722175_195	60.2
pep1-nc-RBM26-AS1	ENST00000456602_79422136_79422246_111	60.2
pep1-nc-AC093928.1	ENST00000656348_57299055_57299264_210	60.2
pep2-nc-AC099344.3	ENST00000656821_11406110_11406229_120	60.2
pep1-nc-ZFAS1	ENST00000371743_49290607_49290711_105	60.6
pep-nc-AC023509.1	ENST00000547717_53455939_53467409_723	60.8
pep2-nc-AC010883.2	ENST00000669652_43130783_43130866_84	61.1
pep1-nc-AL096869.3	ENST00000567837_90490172_90490243_72	61.3
pep1-nc-PCAT19	ENST00000651572_41500737_41501098_258	61.4
pep2-nc-PVT1	ENST00000668619_128047554_128047724_171	61.4
pep2-nc-AC027045.3	ENST00000635215_9808841_9809020_180	61.5
pep1-u-EIF3L	ENST00000412331_37848872_37849033_162	61.9
pep-nc-DACT3-AS1	ENST00000500689_46661564_46661743_180	62.2
pep-nc-RN7SKP197	ENST00000517077_119631237_119631371_135	62.3
pep1-nc-AC048382.1	ENST00000542197_84612061_84614515_192	63.2
pep-nc-AC102941.1	ENST00000567616_29610123_29610308_186	63.2
pep2-nc-CCDC26	ENST00000630386_128905044_128918946_273	63.3
pep-nc-AC016590.1	ENST00000586324_37267452_37267685_234	63.6
pep1-nc-LINC01936	ENST00000670470_30360306_30360452_147	63.6
pep-nc-RN7SL479P	ENST00000472003_44117220_44117297_78	64.3
pep2-nc-AIRN	ENST00000601203_160005494_160005598_105	64.5
pep1-nc-AC099329.1	ENST00000449063_42805202_42805309_108	64.8
pep-nc-CARD8-AS1	ENST00000602172_48255727_48255792_66	64.8
pep-nc-AL139424.3	ENST00000641619_10382366_10382476_111	65.8
pep1-nc-UXT-AS1	ENST00000658400_47692022_47692120_99	66.2
pep1-nc-AC072062.1	ENST00000626134_215042271_215075630_246	66.7
pep1-nc-LINC01220	ENST00000665235_75297773_75297937_165	67.4
pep1-nc-PAXBP1-AS1	ENST00000653345_32745978_32746493_516	67.9
pep1-nc-ZBED3-AS1	ENST00000511547_77146485_77146562_78	68.5
pep2-nc-AC021237.1	ENST00000504175_109315249_109315407_159	68.8
pep-nc-AL356489.4	ENST00000654908_33730361_33730468_108	69.7
pep1-nc-AL391152.1	ENST00000554160_57066609_57066695_87	70.2
pep2-nc-AC092296.1	ENST00000587018_36378702_36378773_72	70.2
pep14-nc-HELLPAR	ENST00000626826_102330263_102330352_90	70.6

pep-nc-AL139095.5	ENST00000650292_7259064_7259156_93	71.9
pep3-nc-AL359924.1	ENST00000450451_237926878_237927552_675	72.1
pep1-nc-LHX1-DT	ENST00000621428_36933362_36933439_78	72.2
pep1-nc-LINC01726	ENST00000624692_21610938_21611054_117	72.2
pep2-nc-FIRRE	ENST00000657242_131824110_131824238_129	74.3
pep1-nc-ALG14-AS1	ENST00000451611_95062184_95067268_231	74.9
pep-nc-AC131944.1	ENST00000662475_87989362_87989460_99	76.5
pep-nc-AL359880.1	ENST00000621879_46717492_46717608_117	76.6
pep2-nc-AC003986.2	ENST00000419944_19113815_19113976_162	78.1
pep-nc-AL356441.1	ENST00000452618_165598543_165598614_72	78.9
pep1-nc-LINC01719	ENST00000452883_146057763_146057837_75	84.7
pep-alt-ARMC1	ENST00000458464_65605508_65627377_285	85.5
pep-nc-LINC01425	ENST00000430060_21748560_21748658_99	85.8
pep1-nc-OLMALINC	ENST00000654233_100410237_100410851_615	89.1
pep2-nc-AC040168.1	ENST00000657483_55984182_55984370_189	90.2
