# Supplementary material for: Comprehensive discovery and functional characterization of the noncanonical proteome
Source: Cell Res. 2025 Jan 10;35(3):186–204. doi: 10.1038/s41422-024-01059-3 (PMC11909191; doi:10.1038/s41422-024-01059-3)
Supplement: Supplementary file 25 — Table S17 [file 41422_2024_1059_MOESM25_ESM.pdf]

Supplementary Information, Table S17. The predicted and validated subcellular localization of peptides

The table presents information on 34 peptides predicted to have specific subcellular localization in the peptide-protein interaction network. Cells shaded yellow indicate that the localization has been experimentally validated.

Pep_name	ORF_ID	Predicted localization
pep-nc-ZNF436-AS1	ENST00000335648_23370095_23370370_276	Mitochondrion
pep-nc-OGFRP1	ENST00000332965_42277406_42277705_300	Cytoplasm
pep-nc-AP002807.1	ENST00000526897_68052329_68053448_309	Endoplasmic reticulum
pep5-nc-TRHDE-AS1	ENST00000667465_72251783_72251968_186	Lysosome
pep3-nc-PCBP1-AS1	ENST00000442326_70094079_70102898_471	Nucleus
pep2-nc-LINC00271	ENST00000664629_135498552_135498815_264	Mitochondrion
pep2-nc-FAM230A	ENST00000624459_18492327_18492986_660	Nucleus
pep2-nc-AIRN	ENST00000601203_160005494_160005598_105	Cytoplasm
pep2-nc-AC027045.3	ENST00000635215_9808841_9809020_180	Mitochondrion
pep1-nc-OLMALINC	ENST00000654233_100410237_100410851_615	Mitochondrion
pep-nc-PAXBP1-AS1	ENST00000653345_32745978_32746493_516	Mitochondrion
pep-nc-MAPT-IT1	ENST00000624111_45897063_45897386_324	Cytoplasm
pep-nc-LINC02183	ENST00000637822_54545356_54546113_222	Mitochondrion
pep-nc-LHX1-DT	ENST00000621428_36933362_36933439_78	Nucleus
pep-nc-AL391152.1	ENST00000554160_57066609_57066695_87	Mitochondrion
pep-nc-AL139095.5	ENST00000650292_7259064_7259156_93	Mitochondrion
pep-nc-AC023509.1	ENST00000547717_53455939_53467409_723	Nucleus
pep-nc-AC009053.3	ENST00000566506_74422164_74434472_309	Mitochondrion
pep-nc-AC006213.1	ENST00000586860_43999143_44002809_276	Mitochondrion
pep-alt-ARMC1	ENST00000458464_65605508_65627377_285	Cytoplasm
pep3-nc-AL359924.1	ENST00000450451_237926878_237927552_675	Mitochondrion
pep2-nc-AC021237.1	ENST00000504175_109315249_109315407_159	Nucleus
pep1-nc-PCAT19	ENST00000651572_41500737_41501098_258	Mitochondrion
pep1-nc-AC127496.3	ENST00000576032_80940472_80941938_345	Mitochondrion
pep1-nc-AC078881.1	ENST00000668108_177599309_177599515_207	Cytoplasm
pep1-nc-AC006504.5	ENST00000585917_27797856_27806018_735	Nucleus
pep-nc-LINC02482	ENST00000667300_6658375_6658683_309	Golgi apparatus
pep-nc-AL359880.1	ENST00000621879_46717492_46717608_117	Golgi apparatus
pep-nc-AL356489.4	ENST00000654908_33730361_33730468_108	Mitochondrion
pep-nc-AL133313.1	ENST00000663643_68693906_68694295_390	Cytoplasm
pep-nc-AC093928.1	ENST00000656348_57299055_57299264_210	Endoplasmic reticulum
pep-nc-AC046158.1	ENST00000563408_78496193_78496351_159	Lysosome
pep2-nc-AC010300.1	ENST00000600671_23325794_23326129_336	Endoplasmic reticulum
pep1-nc-LINC02175	ENST00000571219_25068280_25068537_258	Lysosome
