# Supplementary material for: Comprehensive discovery and functional characterization of the noncanonical proteome
Source: Cell Res. 2025 Jan 10;35(3):186–204. doi: 10.1038/s41422-024-01059-3 (PMC11909191; doi:10.1038/s41422-024-01059-3)
Supplement: Supplementary file 26 — Table S18 [file 41422_2024_1059_MOESM26_ESM.pdf]

Supplementary Information, Table S18. Sequences of Oligonucleotides for Knock out/Knock In and qPCR

This table presents information on all primers utilized in this study.

sORF KO/KI		
**Pep_name**	**ORF_ID**	**sgRNA sequence**
pep1-nc-OLMALINC	ENST00000654233_100410237_100410851_615	CCTAGGAACCCAACTTACAA
pep-nc-AC005005.3	ENST00000609017_31082256_31082981_726	TCAGTTTGCCTGAAATCCAG
pep2-nc-PRKG1-AS1	ENST00000658196_52291671_52292339_669	ACTCATCATCACTGGATCAC
pep1-nc-FAM230A	ENST00000624001_18425006_18489902_441	GAGGCCAGTCAACGTGAGGG
pep2-nc-CDKN2B-AS1	ENST00000651588_22012155_22012412_258	ATTTGCCAGCAAACAGCTGG
pep-nc-FAM215A	ENST00000588043_43917320_43917652_333	AGCCGCTGCTTCTTCAGCCA
pep1-nc-PPM1F-AS1	ENST00000458178_21943911_21944171_261	GCCACCTGCTCCTCTCAAGA
pep-nc-ZNF436-AS1	ENST00000335648_23370095_23370370_276	CCTCGGATCGACTTTAGATG
pep-nc-LINC02482	ENST00000667300_6658375_6658683_309	TGATGCTTTACTACTCCCAA
pep-nc-AL669970.3	ENST00000435284_134544950_134545183_234	GGAGAAGGAATGAGGGTGAG
pep1-nc-LINC00343	ENST00000415294_105707153_105707461_309	CCCCATCTCATCAAGTAAAA
pep5-nc-TRHDE-AS1	ENST00000667465_72251783_72251968_186	AATTAAGCAACCAATCAGAA
pep-nc-AC018866.1	ENST00000421820_120709369_120710510_357	CAGCTAGAAGAAGTGCATTT
pep1-nc-PCAT19	ENST00000651572_41500737_41501098_258	TTTCAGCAGCTTGTCAGGAA
pep2-nc-LINC00271	ENST00000664629_135498552_135498815_264	CTAGGAGAGTATGTTTAGAA
pep-nc-OGFRP1	ENST00000332965_42277406_42277705_300	ACTTGTTCCCTCCTACCGCA
pep2-nc-AC027045.3	ENST00000635215_9808841_9809020_180	TCAGTATCAGAACGATATCC
pep-nc-AC113615.2	ENST00000669245_6184338_6184649_312	ACACTGGCGCTCAGGGTGGA
pep2-nc-AC073611.1	ENST00000676940_53295568_53299342_462	CTATGTAGAGAAGACAGCTG
pep3-nc-PCBP1-AS1	ENST00000442326_70094079_70102898_471	CTGTCCAAACTACTGGCTGG
pep2-nc-FIRRE	ENST00000657242_131824110_131824238_129	GTCTGACATACAAGGTAACT
pep2-nc-BX255925.1	ENST00000566954_137294438_137294557_120	GGGAGGCATCCTTGAAGAAG
pep14-nc-HELLPAR	ENST00000626826_102330263_102330352_90	GAGACTGACTGCTTAATCAT
pep2-nc-AIRN	ENST00000601203_160005494_160005598_105	AAGGGAGTGACCCAGCCGCA
pep1-nc-ZFAS1	ENST00000371743_49290607_49290711_105	CAAGTTATCTCAAAACCTAG
pep-nc-LINC01726	ENST00000624692_21610938_21611054_117	TTCATGCATCATGAAGCAGA
pep-nc-MEF2C-AS2	ENST00000659116_88766292_88766399_108	TTTAATCACTGCATAAGAAA

sORF qPCR	
**Gene**	**Primer sequence**
GAPDH-F	AAGGTGAAGGTCGGAGTCAA
GAPDH-R	AATGAAGGGGTCATTGATGG
U6-F	CTCGCTTCGGCAGCACA
U6-R	AACGCTTCACGAATTTGCGT
ZNF436-AS1-F	TCCAAAACCTGAGAGACCGC
ZNF436-AS1-R	AAGAGGGTGGGAGTCCCTAA
TRHDE-AS1-F	TTGGAAAGCGTCTTGCTTGC
TRHDE-AS1-R	GCGAAAGCCCTAACTTCCCT
OLMALINC-F	AATTCAACAACGCTTCATGC
OLMALINC-R	GAGGTCCTTCACTTCCCTTG
AC027045.3-F	TTGTGTACATCATGTGGGCC
AC027045.3-R	CACCGCGTAGAGTTCTTCCA
LINC01936-F	TACAAAGTGCACAGAGTTGT

LINC01936-R	TTGAGGATGCAAAGATGCTT
AL050309.1-F	CGGCCCTAGTCGGTGCCCAT
AL050309.1-R	ATTCCAGATACATTGTTCCT
LINC02175-F	CACTGGCACTCTTGTCCAGA
LINC02175-R	TTCTGCAGAACAGGTGAAAG
AL589987.2-F	GGCTAAGGAGACCCACACAC
AL589987.2-R	AGTTGTACGTTTCTGATCCA
LINC01613-F	TAAGTCTTCTCCTGGATCTA
LINC01613-R	GAAAGATTACACCCCGCCCC
AC023509.1-F	GCTGGACCCACTAATGCCAT
AC023509.1-R	AACCAGCAGGATGCTGGATA
MAP3K20-AS1-F	CGCGGCTTCATGAAGTCGCA
MAP3K20-AS1-R	ATTCTCCCTCCCAGGCATGG
AP000919.1-F	GCTCCTGGCTTCACATGAAC
AP000919.1-R	CACGTCTTGGAAACAGAGAC
AL391832.3-F	GTGCCGTCTGAACAGACATC
AL391832.3-R	ATGGCAGTGACTGGATGCTG
LINC01435-F	CCCTGTGCTTATCTAGGATT
LINC01435-R	TGGCACCATTGAAGCTTTTT
ZBTB20-AS1-F	CTGGACTACAAGTGCACACC
ZBTB20-AS1-R	AAGAGATTTTCACGCTGGGC
AC012101.2-F	CCAGGAGCAAGAGCCTTGTG
AC012101.2-R	ATGGGAGTTTCGCATTTTGC
SCHLAP1-F	TGGGAGGAACGAACAACTCC
SCHLAP1-R	TATTGATGGCACCTGAGGAC
AC138028.5-F	GTGTGGATGGGTCTCCAGGT
AC138028.5-R	CAGGTGAAACGCTCCAGGGA
AC072062.1-F	CTGTCACCCAGGCCAGAGAG
AC072062.1-R	TTGTGATGGATGGATGGCCC
AC006504.5-F	CTGGAGACAGAGCTCTCCCA
AC006504.5-R	GGATGCCCAGGGACTGGTGA
AL359924.1-F	GTGCAAAGCTGGCTTTGCTG
AL359924.1-R	TCTCCAGGGAGGAGGAAGAT
AC096667.1-F	CTGCACCCCTCTGTGATTCG
AC096667.1-R	TCCAGGAGTCTCCCACTTGG
AC005562.1-F	TTACAAGCCCGGAAGCAGCA
AC005562.1-R	GAACAAAGCACACCTCAGCC
MAPT-IT1-F	GAGCCTGGGTACTGCGAGGC
MAPT-IT1-R	CGAAATCGCAGAAGCGCGGA
AL589987.2-F	GGAGACCCACACACCAGTGA
AL589987.2-R	CCAGAGTTGCTGTTGCTAGT
LINC01608-F	GGAGGCCTCACAATCCTGGT
LINC01608-R	AGAAGTGGTAAGAGTGGGCC
AL359924.1-F	GTGCAAAGCTGGCTTTGCTG
AL359924.1-R	CGTGGCCATCTCCTTCTCGA
AC096667.1-F	CTGCACCCCTCTGTGATTCG
AC096667.1-R	GAGTCTCCCACTTGGCGGTG
AC005562.1-F	TACAAGCCCGGAAGCAGCAC
AC005562.1-R	CTGAATCCACACAAACACAGG
MFF-DT-F	CACCTGCAGAAGACGCTCCT
MFF-DT-R	TTGGGAGGCTGAGGCAGGAG
BLACE-F	CTGGCTGCACATCTGTCTGG
BLACE-R	TCGCAGGAGGATGGCGTAAG
AC010300.1-F	CCTGCTGCCAGAGTCATCAT
AC010300.1-R	CTTTGCTGGGGGAAGGGGAT

CTBP1-AS-F	GGAGAGGGCAGGGAGCAGAG
CTBP1-AS-R	TTGTCGTCGTCCGTCCTGTG
CCDC26-F	GTGACCTTCTCAGACCCTGT
CCDC26-R	GCTGGCAGCCACTCTCAGAG
AC009053.3-F	CTGGCGTGATCCCACCAGAG
AC009053.3-R	AATCCCCAGGTGTCGAGGGA
AL353593.1-F	GTGTCTTGTCATCCGGCGTC
AL353593.1-R	CTGAGGGTCCGAGGTGAGTG
MUC20-OT1-F	GTGCCTCTGCACATGGTGTC
MUC20-OT1-R	CCAACAGTCCACTCCTTCCT
AC012508.1-F	GCAGATGGCACTGGTCTCAG
AC012508.1-R	ACTGGAATCCCAACTCACGC
LINC02346-F	AATGAAGCCATGGCCCCTCA
LINC02346-R	CCACATGGCACTGGTCTCAG
CASC19-F	TTCACTTCTGCCAGTGTCCC
CASC19-R	TGCCAGATGCTTGGGATAGC
LINC02876-F	ACTCCTCAGCTTCAGATGGT
LINC02876-R	AGGAGCTCCTCTCAGAGGTG
AC244502.1-F	AGGAAGCTCCAAACTTTCCC
AC244502.1-R	TCCTTGCCTTCTGCCATGAT
SPACA6P-AS-F	GTCACAGGGACCACTGGTTC
SPACA6P-AS-R	CACTTCTGACGTTACGTCGG
AC068418.2-F	ATGTTCCCTCCTGCTCCCCG
AC068418.2-R	CAAGGGAGGTGAGGAGAGGG
