# Supplementary material for: Comprehensive discovery and functional characterization of the noncanonical proteome
Source: Cell Res. 2025 Jan 10;35(3):186–204. doi: 10.1038/s41422-024-01059-3 (PMC11909191; doi:10.1038/s41422-024-01059-3)
Supplement: Supplementary file 27 — Table S19 [file 41422_2024_1059_MOESM27_ESM.pdf]

Supplementary Information, Table S19. The correlation between clinicopathological parameters and peptide expression in gastric cancer.

The table displays the expression levels of OLMALINC, TRHDE-AS1, AC027045.3, and ZNF436-AS1 in patients from the Second Affiliated Hospital, School of Medicine Zhejiang University cohorts, in relation to age, gender, invasive depth, lymph node metastasis and TNM stage.

	TRHDE-AS1 **expression**		***P****
	**Low, n(%)**	**High, n(%)**	
**Age**			
< 45	7(43.8)	9(56.2)	0.571
≥45	29(51.8)	27(48.2)	
**Gender**			
Male	24(57.1)	18(42.9)	0.151
Female	12(40.0)	18(60.0)	
**Invasive depth**			
T1-T2	15(71.4)	6(28.6)	0.020
T3-T4	21(41.2)	30(58.8)	
**Lymph node metastasis**			
Absent	18(43.9)	23(56.1)	0.234
Present	18(58.1)	13(41.9)	
**TNM Stage**			
I-II	20(64.5)	11(35.5)	0.032
III-IV	16(39.0)	25(61.0)	
**P* values determined by Chi-square test using SPSS 25.0. All statistical tests were two-sided.			

	AC027045.3 **expression**		***P****
	**Low, n(%)**	**High, n(%)**	
**Age**			
< 45	10(62.5)	6(37.5)	0.257
≥45	26(46.4)	30(53.6)	
**Gender**			
Male	18(42.9)	24(57.1)	0.151
Female	18(60.0)	12(40.0)	
**Invasive depth**			
T1-T2	10(47.6)	11(52.4)	0.795
T3-T4	26(51.0)	25(49.0)	
**Lymph node metastasis**			
Absent	17(41.5)	24(58.5)	0.096
Present	19(61.3)	12(38.7)	
**TNM Stage**			
I-II	15(48.4)	16(51.6)	0.812
III-IV	21(51.2)	20(48.8)	
**P* values determined by Chi-square test using SPSS 25.0. All statistical tests were two-sided.			

	OLMALINC **expression**		***P****
	**Low, n(%)**	**High, n(%)**	
**Age**			
< 45	8(50.0)	8(50.0)	1.00
≥45	28(50.0)	28(50.0)	
**Gender**			
Male	19(45.2)	23(54.8)	0.339
Female	17(56.7)	13(43.3)	
**Invasive depth**			
T1-T2	13(61.9)	8(38.1)	0.195
T3-T4	23(45.1)	28(54.9)	
**Lymph node metastasis**			
Absent	19(46.3)	22(53.7)	0.475
Present	17(54.8)	14(45.2)	
**TNM Stage**			
I-II	12(38.7)	19(61.3)	0.096
III-IV	24(58.5)	17(41.5)	
**P* values determined by Chi-square test using SPSS 25.0. All statistical tests were two-sided.			

	***ZNF436-AS1* expression**		***P****
	**Low, n(%)**	**High, n(%)**	
**Age**			
< 45	11(68.8)	5(31.2)	0.089
≥45	25(44.6)	31(55.4)	
**Gender**			
Male	20(47.6)	22(52.4)	0.632
Female	16(53.3)	14(46.7)	
**Invasive depth**			
T1-T2	12(57.1)	9(42.9)	0.436
T3-T4	24(47.1)	27(52.9)	
**Lymph node metastasis**			
Absent	18(43.9)	23(56.1)	0.234
Present	18(58.1)	13(41.9)	
**TNM Stage**			
I-II	17(54.8)	14(45.2)	0.475
III-IV	19(46.3)	22(53.7)	

**P* values determined by Chi-square test using SPSS 25.0. All statistical tests were two-sided.
